# NMR Analysis of Extra Virgin Olive Oil of the Epirus Region of Greece with Emphasis on Selected Phenolic Compounds

**DOI:** 10.3390/molecules29051111

**Published:** 2024-03-01

**Authors:** Theodoros Tsolis, Dimitra Kyriakou, Evangelia Sifnaiou, Dimitrios Thomos, Dimitrios Glykos, Constantinos G. Tsiafoulis, Achilleas Garoufis

**Affiliations:** 1 Laboratory of Inorganic Chemistry, Department of Chemistry, University of Ioannina, 45110 Ioannina, Greece; d.kyriakou@uoi.gr (D.K.); e.sifnaiou@uoi.gr (E.S.); d.thomos@uoi.gr (D.T.); d.glykos@uoi.gr (D.G.); 2 NMR Centre, Laboratory of Analytical Chemistry, Department of Chemistry, University of Ioannina, 45110 Ioannina, Greece; ctsiafou@uoi.gr; 3 School of Science & Technology, Hellenic Open University, 26335 Patras, Greece; 4 Institute of Materials Science and Computing, University Research Centre of Ioannina (URCI), 45110 Ioannina, Greece

**Keywords:** olive oil, phenolics, Epirus region, multi-suppression NMR, health claim, effect factors

## Abstract

Extra virgin olive oil (EVOO) is recognized for its numerous health benefits, attributed to its rich phenolic components. NMR has emerged as a prevalent technique for precisely identifying these compounds. Among Mediterranean countries, Greece stands as the third-largest producer of olives, with the Epirus region notably advancing in olive cultivation, contributing significantly to the dynamic growth of the region. In this study, an NMR method was employed based on the acquisition of a ^1^H NMR spectrum along with multiple resonant suppression in order to increase the sensitivity. Using the above method, 198 samples of extra virgin olive oil, primarily sourced from the Epirus region, were analyzed, and both the qualitative and quantitative aspects of the phenolic compounds were obtained. In addition, we examined the effects of various factors such as variety, harvest month, and region origin on the phenolic compounds’ concentration. The results revealed an average total phenolic content of 246 mg/kg, closely approaching the EU health claim limit of 250 mg/kg. Approximately 15% of the samples were confidently characterized as high-phenolic olive oil. The highest concentrations were observed in the Thesprotia samples, with several Lianolia varieties exceeding the total phenolic content of 400 mg/kg. Statistical tests demonstrated a significant influence of the olive variety and the month of fruit harvest on phenolic component concentration, followed by the region of origin. A very strong correlation was noted between the total phenolics content and the levels of oleocanthal and oleacein, with a correlation coefficient (r) of 0.924. Upon optimization of all factors affecting olive oil quality, the majority of the EVOOs from the Epirus region have the potential to be characterized as high in phenolic content.

## 1. Introduction

Extra virgin olive oil (EVOO) is a significant component of the Mediterranean diet, which has garnered noteworthy scientific attention due to its protective effects on human health [1]. EVOO predominantly contains monounsaturated fatty acids, with the unsaponified portion not exceeding 2% [2]. Despite its relatively low percentage, the phenolic compounds within this fraction are of paramount importance, as they are responsible for its oxidative stability, its functional activity, and its unique organoleptic properties [3]. These compounds, together with α-tocopherol and β-carotene, offer significant benefits to humans, as they exhibit anti-inflammatory and robust antioxidant properties, contributing to a reduction in morbidity and a slowdown in the progression of diseases related to the heart, nervous system, and cancer [4]. In fact, European Regulation 432/2012 has classified olive oils based on their impact on health, taking into account their content of these substances. This regulation explicitly states that blood lipids are shielded from oxidative stress when the phenolic content in olive oil reaches at least 250 mg/kg [5]. Additionally, Diamantakos et al. have introduced the term “high phenolic olive oil” for oils containing phenolic levels surpassing 500 mg/kg, a threshold that exceeds the European Regulation 432/2012 limit (250 mg/kg), for at least 12 months following bottling. In the same document, they also refer to the classification of olive oils as “exceptionally rich” in phenolic components, a classification that applies to those boasting a phenolic content exceeding 1200 mg/kg [6].

**Scheme 1:**
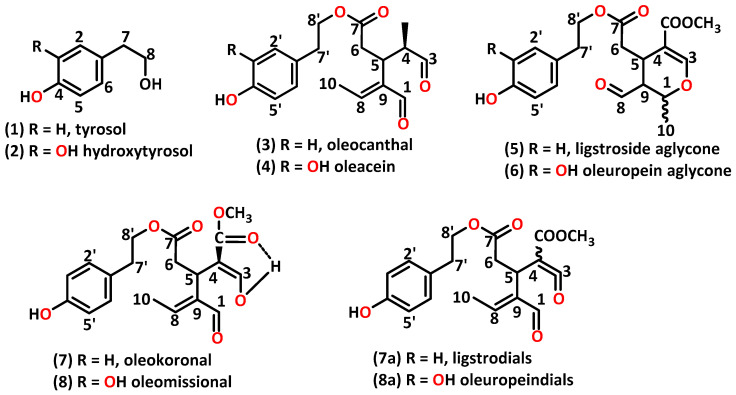
The structures of the studied phenolic compounds.

Secoiridoids (SECs) and lignans represent the two categories of aromatic alcohols found in olive oils. Within the SEC group, there are tyrosol (TYR) (**1**) and its esterified derivatives, including oleocanthal (**3**), the monoaldehyde form of ligstroside aglycone (**5**), oleokoronal (**7**), and the two isomers of ligstrodials (**7a**). Additionally, there are hydroxytyrosol (HTYR) (**2**) and its corresponding derivatives, such as oleacein (**4**), the monoaldehyde form of oleuropein aglycone (**6**), oleomissional (**8**), and the two isomers of oleuropeindials (**8a**) (Scheme 1) [7]. Oleocanthal exhibits anticancer [8], antimicrobial (against Helicobacter pylori) [9], and anti-inflammatory activity similar to ibuprofen [10]. Furthermore, it has been reported to inhibit the progression of Alzheimer’s disease [11]. The HTYR dialdehydic derivative oleacein possesses antioxidant [12], anti-inflammatory [13], and anti-atherosclerotic [14] properties along with anti-breast cancer attributes [12] and neuroprotective activity [15]. Not only anti-breast cancer properties but also significant protection against cardiovascular diseases has been reported to be exhibited by oleuropein aglycone [16]. Recently, the role of secoiridoids for the classification of EVOO has been reported [17].

The quality of olive oil and its polyphenol content are influenced by various factors, including the region of origin, the olive tree variety, the harvest season, and the procedures that have been used for the extraction of the olive oil [1,18,19]. Several analytical techniques and methods, such as gas chromatography (GC), UV spectroscopy, liquid chromatography (LC), mass spectrometry (MS), and nuclear magnetic resonance spectroscopy (NMR) have been employed to assess these factors [17,20,21,22]. NMR spectroscopy offers “high-throughput” structural information, rendering it a potent technique for both qualitative and quantitative analysis of small molecules in complex mixtures, and for this reason, it has been used successfully in the field of food science for over two decades [12,23,24]. Nuclei like ^1^H, ^13^C, and ^31^P have been extensively utilized in NMR studies of olive oils [25,26,27]. Furthermore, NMR offers an advantage in terms of the range of experiments that can be conducted using the same sample. Two-dimensional homonuclear and heteronuclear NMR experiments, as well as maximum-quantum (MaxQ) NMR, represent some of the approaches that have been employed for the analysis of olive oils and their polyphenols [28,29,30]. NMR methods that have the ability to enhance minor components’ signals through the suppression of major ones have been successfully applied in the analysis of several foods, such as milk samples [24]. In addition, multiple signal suppression has been successfully used in olive oil samples without requiring any sample pretreatment (extraction and/or purification) [31]. The determination of polyphenols through chromatographic methods necessitates the construction of calibration curves using standard solutions of the compounds of interest. However, these standards may be either commercially unavailable or too expensive, require significant effort to isolate, or are unstable [32]. Quantitative ^1^H nuclear magnetic resonance (qNMR) spectroscopy enables the quantification of molecules in multi-component samples without the need for standard compounds [33,34]. Thus, qNMR, which enhances the advantages of the NMR technique, serves as a complement to chromatographic methods for accurately quantifying compounds within a complex food matrix [35].

One of the main agricultural products of the Mediterranean countries, mainly Spain, Italy, and Greece, is EVOO [36]. Greece is the third-largest producer in the Mediterranean, yielding approximately 400,000 tons annually [27]. The regions of Crete, as well as the southern parts of the Peloponnese, particularly Laconia and Messinia, are renowned for their olive oil production. As a result, the majority of NMR-based studies related to Greek olive oils have primarily focused on samples from these regions [27,30,37,38,39,40,41,42,43], and there is a limited number of publications with studies of samples from the Ionian islands [44,45]. Over the past decade, the region of Epirus, situated in the northwest of Greece, has made a significant entrance onto the domestic olive-growing scene. Olive oil is produced in three of the four provinces within this area, called Arta, Preveza, and Thesprotia. Common olive varieties in this region include Lianolia, Amfissis (Konservolia), Koroneiki, Kalamon, and Mesokarpos (the local variety of Thesprotia). However, the quality and polyphenol concentration of Epirus olive oil has not been studied extensively using NMR methods. Up to date, there is a limited number of NMR studies; two conducted by Karkoula et al., incorporating a small subset of samples from the Epirus region (3 out of 175 and 340 total samples, respectively), and a recently published study by Tsiafoulis et al. [17,46,47].

Herein, we report on the NMR study of the phenolic content of EVOO from the Epirus region, employing both 1D ^1^H NMR methods and multi-suppression NMR experiments. Additionally, we assessed how the region, variety, and harvest month affect the phenolic content. For comparison reasons, with the above-mentioned methods, we also analyzed the phenolic content of EVOO from other selected regions of Greece (Messinia, Kerkyra, Zakynthos, and Crete).

## 2. Results and Discussion

### 2.1. NMR Analysis

**Figure 1:**
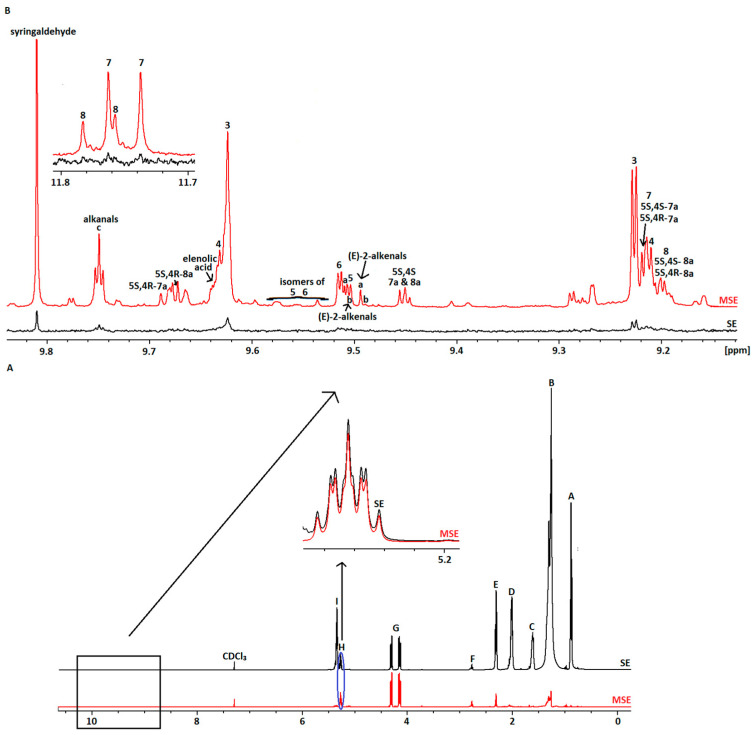
(**A**) Comparison of SE and MSE spectra of selected Epirus olive oil; (**Β**) Aldehydic region of the SE and MSE spectra, with assignment of the studied phenolic compounds.

Figure 1A displays the spectrum of the multi-suppression experiment (MSE) in comparison with the standard spectrum (SE) of an olive oil sample: the Lianolia variety from Parga (Preveza, Greece). The suppression was performed in the proton signals related to oleic acyl groups that showed the highest intensities (0.80–5.50 ppm). The following six peaks in particular were suppressed: 0.88 ppm (three frequencies), 1.22–1.42 ppm (four frequencies), 1.63 ppm (three frequencies), 2.02 ppm (three frequencies), 2.34 ppm (two frequencies), and 5.33 ppm (two frequencies). The scaling of these spectra was performed with the dd signals (**G**) of the -CH_2_ glyceryl group of triglycerides left unsuppressed. While the impact of suppressing the lipid signals is clearly evident, peaks in their vicinity remain unaffected (see Figure 1A, signal **H**). The absence of these peaks results in the appearance of the phenolic signals (minor components), as illustrated in Figure 1B. The chemical shifts of the major (**A**–**I**) and minor compounds are shown in Table S1 in the Supplementary Materials and are assigned according to the literature [17,48].

### 2.2. Direct Quantification of Phenolic Content

**Figure 2:**
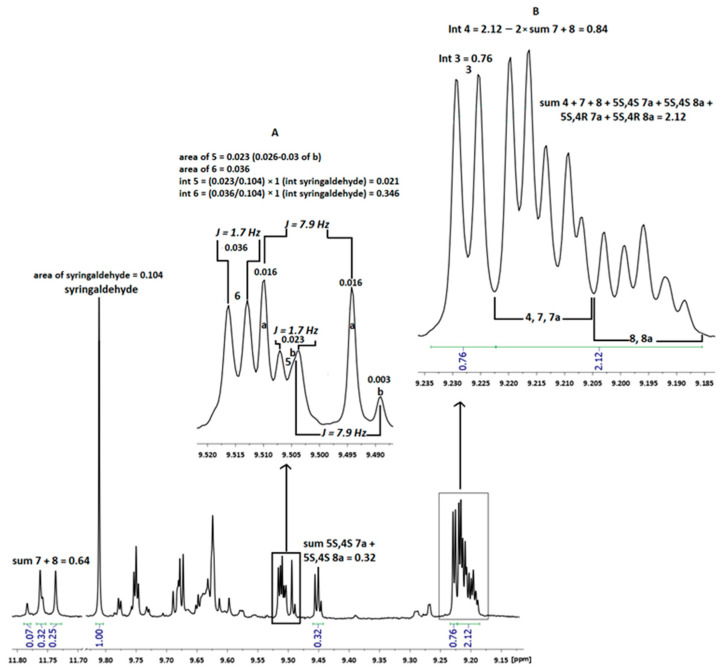
Part of the noesygppr1d spectrum of EVOO from Vlaxerna (Arta), showing the ratio of oleokoronal (**7**) and oleomissional (**8**) with 5S,4S ligstrodial (**7a**) and 5S,4S oleuropeindial (**8a**) (2:1), respectively; (**A**) The coupling constants of the peaks in the magnified area; (**B**) The calculation of oleacein (**4**) peak integration. Area = Value for the deconvolution results; Int = Integral of each component.

QNMR is a suitable technique and has been applied to the quantification of phenolics in other olive oil samples as well [1,46,47,49]. The quantitative analysis refers to the tyrosol and hydroxytyrosol derivatives, and the sum of these corresponds to the total phenolics. The method is based on the integration of the aldehydic protons of oleocanthal (**3**) (9.23 ppm), oleacein (**4**) (9.21 ppm), ligstroside (**5**) and oleuropein (**6**) aglycones (9.50 ppm and 9.52 ppm, respectively) as well as of the enolic protons of oleokoronal (**7**) (11.75 ppm) and oleomissional (**8**) (11.77 ppm). Oleokoronal and oleomissional pertain to the mixture of oleokoronal with the two isomers of ligstrodials (**7a**) and of oleomissional with the two isomers of oleuropeindials (**8a**), respectively. It has been reported that they are present in olive oil in equilibrium at a stable ratio of 2:1:1 when the spectrum is recorded in CDCl_3_ at 298 K [7]. In some cases, the signal of (**4**) (double peak) overlapped with the aldehydic protons of (**7**), (**8**), and the isomers of (**7a**) and (**8a**). In these cases, the amount of (**4**) was estimated by knowing the integration of (**7**) and (**8**) and subtracting it from the total integration of the region of 9.19–9.22 ppm. On the other hand, concerning the peak of (**3**), there was no similar problem in any spectrum, as previously described [46]. Finally, the amounts of (**5**) and (**6**) were calculated using the deconvolution method, a process proposed to determine the contribution of an individual peak to the total area [34]. Based on the coupling constants (*J*) of peaks in the region 9.49–9.52 ppm, we observed that usually the double signal of (**5**) overlapped with the individual peak of **b** and the signal of (**6**) with the peak of **a**. Specifically, through the results of the deconvolution, the values of the areas for (**5**), (**6**), **a**, and **b** were found. As shown in Figure 2A, for (**5**), which overlaps with **b**, its area was calculated after subtracting the value 0.003 (area **b** at 9.489 ppm) from the total area at 9.504–9.507 ppm (0.026). Exactly the same procedure was followed, where necessary, for the calculation of the area of (**6**), subtracting the area of **a**. The integral of these was found by dividing their area by the area syringaldehyde (IS), whose integral was set to 1. Further details are illustrated in Figure 2.

For compounds (**3**), (**4**), (**5**), (**6**), (**7**), and (**8**), molecular masses of 304.34, 320.34, 361.37, 377.37, 362.67, and 378.37 were used, respectively, and for the internal standard (IS), a mass of 182.17 was employed. The mass calculation for each component was conducted based on equation 1, as illustrated in the example below concerning oleocanthal (**3**). Its integral in sample number 74 was determined to be 0.76 (Figure 2B). The ratio Ix/Istd = 0.76/1 = 0.76, the ratio Nstd/Nx = 1 (indicating the same number of resonance nuclei), while the ratio Mx/Mstd = 1.67 (340.34/182.17). The product of the mass of IS, used in each sample, and Pstd is 0.01 × 0.985 = 0.00985. Consequently, the mass of (**3**) for the specific sample was calculated to be 0.76 × 1 × 1.67 × 0.00985 = 0.0125 mg. The mass of 200 μL of EVOO corresponded to 160.80/10^−6^ kg, hence the mass of (**3**) was estimated at 77.75 mg/kg. Employing the same rationale, the masses of the other phenolic components were computed for all samples.

The analysis was conducted within a maximum of 4 months after the production of the olive oil. For this reason, the concentration of hydrolyzed and oxidized phenolic compounds was low and did not affect the total phenolic content of the olive oil [6].

### 2.3. Concentration of Phenolic Compounds

The total phenolic concentration varied between the Epirus samples and those from other regions. In the case of the Epirus olive oils, the amount ranged from 1 mg/kg to 1100 mg/kg (Table 1). The highest concentration of polyphenols was recorded in two samples from Thesprotia, which were of the Kalamon variety. In both of these olive oil samples, oleocanthal (**3**) had the highest concentration among all the studied phenolics, with over 700 mg/kg. The higher content of total phenolics and (**3**) in the Kalamon cultivar has also been reported in other related studies [6,50]. In addition, (**3**) content exceeded that of oleacein (**4**) in the great majority of the olive oil samples. Oleacein (**4**), which is structurally similar to (**3**), had a maximum concentration of 288 mg/kg. The Lianolia variety stood out for its high (**4**) content (Table 2). High (**4**) values were also found in other related studies in Greek olive oils of the specific cultivar, between 19 and 23 studied varieties, respectively [46,47]. The maximum content of ligstroside aglycone (**5**) and oleuropein aglycone (**6**), which are associated with the bitter and pungent taste of olive oil, was estimated to be 134 mg/kg and 80 mg/kg, with averages of 16 mg/kg and 18 mg/kg, respectively. The highest sum of aglycon compounds was observed in olive oil from Thesprotia (Table S2). Finally, the amounts of oleokoronal (**7**) and oleomissional (**8**) ranged from undetected to 130 mg/kg and 79 mg/kg, respectively (Table S3).

Compared with other relevant NMR studies, the phenolic content of Epirus olive oil samples does not differ significantly from that of other Greek regions [47]. In some cases, it is richer in phenolics than other Greek and Italian olive oil samples [27,51]. Additionally, when compared with samples from other regions in this study, Epirus olive oil samples were found to have a higher concentration of phenolic compounds (Table S4).

**Figure 3:**
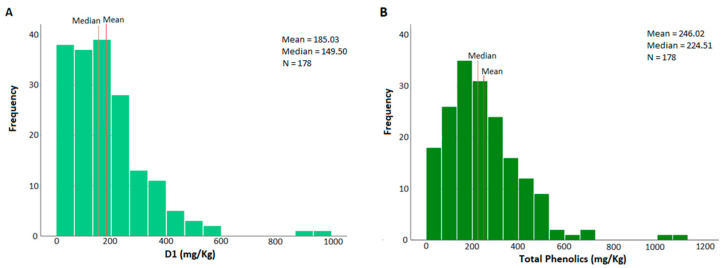
(**A**) The sum of oleocanthal (**3**) and oleacein (**4**) distributions of the analyzed Epirus olive oil samples (N = 178). The red line represents the mean and the brown line the median; (**B**) The total phenolic distributions of the analyzed Epirus olive oil samples (N = 178). The red line represents the mean and the brown line the median.

The distributions of the sum of (**3**) and (**4**), as well as the content of total phenolics, are characterized by high variability, mainly due to the olive variety. Well-known varieties, such as Kalamon or Lianolia, have an increased tendency to produce high amounts of oleocanthal and, therefore, total phenolics [6]. In most samples, D1 is below 400 mg/kg (Figure 3A); however, more than 40% of the samples have a total phenolic content exceeding the EU health claim limit of 250 mg/kg, with the mean very close to it at 246 mg/kg (Figure 3B).

### 2.4. Variety Impact on Phenolic Content

The amounts of the secondary components depend on the olive variety. It has been reported that some varieties primarily yield secoiridoid derivatives, while others produce flavonoids and lignans. Some varieties also exhibit increased levels of specific phenolics that can influence the organoleptic properties of the olive oil produced from them [1].

The Koroneiki variety represents 70% of the total Greek production among 100 recorded varieties [6]. However, in the present study, only 20% of the samples corresponded to this particular variety. This is because the predominant varieties in the region of Epirus are Lianolia (found mainly along the coast of Epirus, particularly in Preveza), Amfissis (in Arta), and, to a lesser extent, Mesokarpos, which is a local olive variety in Thesprotia.

**Figure 4:**
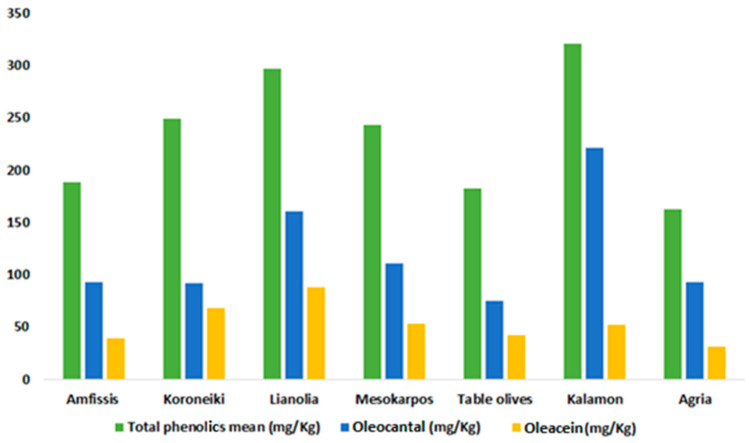
Means of total phenolics, oleocanthal (**3**), and oleacein (**4**) for the studied olive oils in Epirus, categorized by variety. The mean values are expressed in mg/kg.

**Figure 5:**
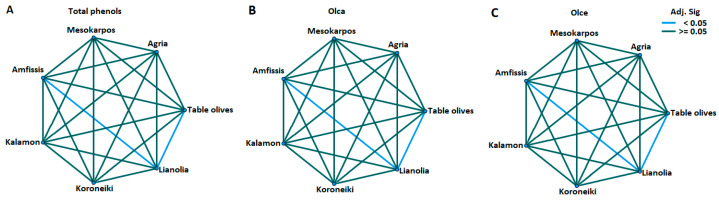
Pairwise comparisons between varieties for (**A**) Average total phenolics; (**B**) Average oleocanthal (**3**); and (**C**) Average oleacein (**4**). Significance values have been adjusted using the Bonferroni correction for multiple tests.

The non-parametric Kruskal–Wallis H test revealed a statistically significant difference in the total phenolic content among the olive varieties (*p* = 0.005). Kalamon had the highest average value, followed by Lianolia and Koroneiki (Table 3 and Figure 4). Notably, the highest total phenolic content of 1100 mg/kg, as shown in Table 1, was also attributed to Kalamon. Statistically significant differences were also observed in the concentration of oleocanthal (**3**) and oleacein (**4**) among the Epirus olive oil samples. Kalamon also exhibited the highest mean in (**3**), followed by Lianolia and Mesokarpos. Conversely, Lianolia had the highest average in (**4**), with one sample from this variety also recording the highest value (Table 1 and Table 3). Pairwise comparisons among the varieties in all three cases indicated differences (*p* < 0.05) between Lianolia and Amfissis as well as the table olive cultivar (Figure 5).

The results of the statistical tests are different for the other phenolic compounds. Kalamon and Mesokarpos varieties displayed the highest ligstroside aglycone (**5**) values. The local Thesprotia variety also stood out with its average oleuropein aglycone (**6**) content, known for its properties against breast cancer and cardiovascular diseases (see Table S5) [16]. In contrast, besides the Koroneiki variety, Amfissis, a cultivar from the Arta region, was found to have a high mean content of oleokoronal (**7**) (Table S6). Statistically significant differences were observed between the Lianolia and table olive varieties for (**5**) and between Koroneiki and Amfissis for (**6**). Koroneiki significantly differed from Kalamon and Lianolia cultivars regarding the average (**7**) and oleomissional (**8**) contents (Figure S1). It is worth noting that, while Kalamon and Lianolia produce large amounts of (**3**) and (**4**), they have lower levels of other phenolic components, such as (**7**) and (**8**) [6].

### 2.5. Harvest Month Impact on Phenolic Content

The selection of the olive oil production month was determined through consultation with local olive mills. Samples resulting from the blending of olive oils from multiple months were excluded from our study. An attempt was made to classify samples by week, but it proved to be unfeasible. The assessment of statistically significant differences was also conducted using the non-parametric Kruskal–Wallis H test due to the non-normal distribution. Table 4 demonstrates a statistically significant difference in Epirus olive oil samples when categorized by harvest month (*p* < 0.001). This indicates that the month of harvest significantly affects the phenolic content of the samples, irrespective of geographical origin, variety, and olive mill. Similar findings have been reported in various relevant studies in the literature [1,19,52,53,54,55,56,57].

**Figure 6:**
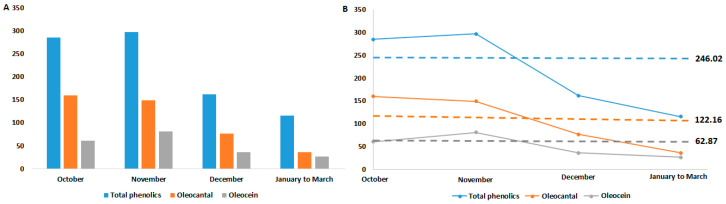
(**A**) Means of total phenolics, oleocanthal (**3**), and oleacein (**4**) for the studied olive oils of Epirus as a function of harvest month; (**B**) Difference in concentration of total phenolics, oleocanthal (**3**), and oleacein (**4**) in relation to harvest month, with the corresponding means marked by a dashed line. Mean values are expressed in mg/kg.

In the initial months of olive harvesting, as well as in the subsequently extracted olive oil, the content of total phenolics, particularly polyphenols such as oleocanthal (**3**) and oleacein (**4**), is high. In contrast, olive oils obtained from ripe olives (December to March) are relatively low in phenolic components (see Figure 6A). The average content of each component is greater in the first two months and decreases from December onward, indicating a decrease in concentration with olive ripeness, but not in a linear fashion (see Figure 6B). While most studies suggest a linear decrease in phenolic components as a function of the harvest month [6,19,52,53,54,55], there are researchers with findings similar to ours, showing an initial increase in concentration, reaching a maximum, and then a decrease during ripening [56,57].

**Figure 7:**
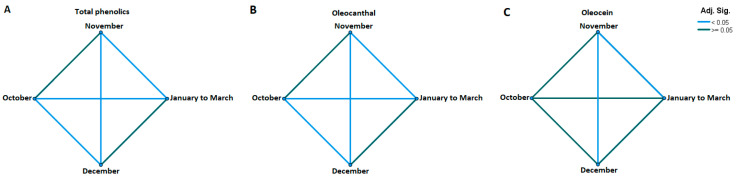
(**A**) Pairwise comparisons between harvest months. (**A**) Average total phenolics; (**B**) Average oleocanthal (**3**); (**C**) Average oleacein (**4**). Significance values have been adjusted using the Bonferroni correction for multiple tests.

Figure 7 supports this observation, with pairwise comparisons revealing a statistically significant difference between the months of October and November and the months of December to March for both total phenolics and (**3**). As for (**4**), the statistically significant difference was limited to the month of November compared with the months of December to March.

Regarding the other components, only ligstroside aglycone (**5**) exhibited a statistically significant difference, with *p* = 0.024, showing a linear decrease in the mean concerning the harvest month (Table S7). Notably, November significantly differed from the months of January to March (Figure S2).

### 2.6. Effect of Prefecture, Altitude, Rainfall, and Average Temperature on Phenolic Content

Many studies have focused on the geographical classification of olive oils and their fingerprinting using various NMR techniques [58]. Therefore, it would be useful to examine whether the prefecture of the Epirus r3egion affects the phenolic content of olive oils. The Epirus region is divided into four prefectures, three of which have olive trees (Arta, Preveza, and Thesprotia).

**Figure 8:**
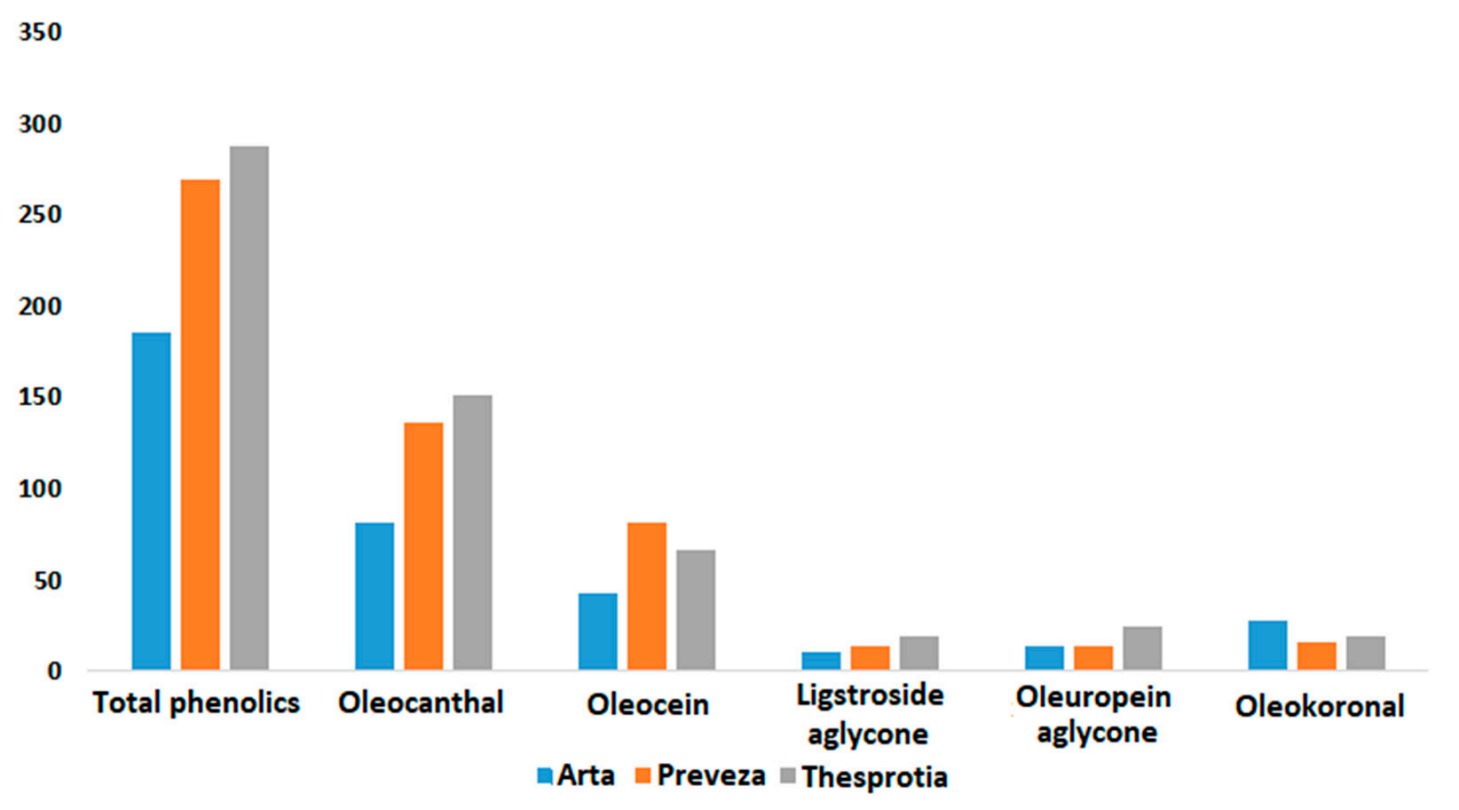
Means of the phenolic compounds and total phenolics for the studied olive oils of Epirus as a function of prefecture. Values of means are expressed in mg/kg.

Table 5 and Table S8 present the results of the Kruskal–Wallis H test and the average phenolic content among prefectures. In all cases, except for oleomissional (**8**), a statistically significant difference between the prefectures was observed (*p* < 0.05). Olive oil samples from Thesprotia exhibited higher mean values of total phenolics and oleocanthal (**3**), whereas Preveza had the highest mean value of oleacein (**4**). These findings are consistent with those in Table 1 and Table 2, where the samples with the highest total phenolics and (**3**) values were from Thesprotia and the (**4**) values were highest in Preveza. Similarly, the examination of the effect of variety on phenolic content revealed a similar pattern, suggesting that the prevailing olive variety in each prefecture may have a more significant influence on polyphenol content than the prefecture itself. This observation holds true for the averages of ligstroside aglycone (**5**), oleuropein aglycone (**6**), and oleokoronal (**7**). The first two are found in high concentrations in Thesprotia, where the Mesokarpos variety is exclusive, while (**7**) is prevalent in Arta, where 50% of samples come from the Amfissis variety. These results are summarized in Figure 8.

Pairwise comparisons based on prefecture revealed statistically significant differences for total phenolics, (**3**), and (**4**) between Arta and both Preveza and Thesprotia (see Figure S3A–C). The same pattern was observed for (**7**), but with the difference that this phenolic component was present in higher concentrations in Arta compared with the other two prefectures (see Figure S3F). As for (**5**) and (**6**), a statistically significant difference was identified only between Thesprotia and Arta (see Figure S3D,E).

In an attempt to determine whether the region truly influences the content of phenolic components or if it is primarily an effect of the predominant variety in each prefecture, a series of climatological factors was examined. Along with the altitude of each area [59], we calculated the average temperature and rainfall values [60], as summarized in Table S9. These calculations resulted in the creation of three new binary variables.

The altitude of each area was grouped into two categories, <150 m (category 1) and >150 m (category 2). We chose a threshold of 150 m to ensure a similar number of samples in each category. The non-parametric Mann–Whitney U test showed no statistically significant difference between the two classes for any phenolic component, including the total (see Table S10). Interpreting this result, we conclude that the altitude of the region of origin of the olive does not significantly affect the phenolic content. This strengthens our hypothesis that the variety is the primary factor determining the concentrations of polyphenols.

**Figure 9:**
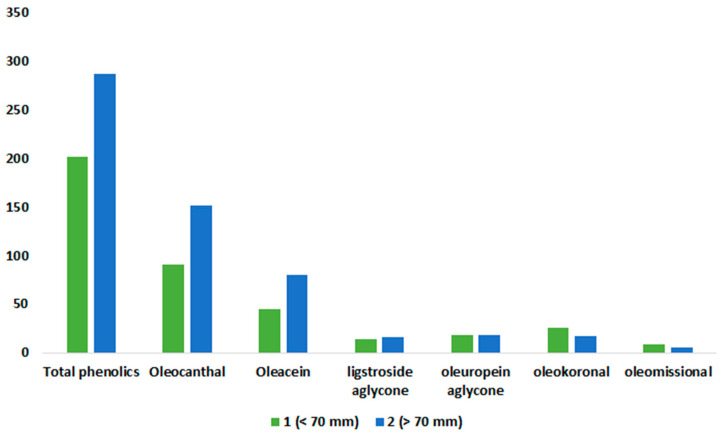
Means of phenolic compounds and total phenolics for the studied olive oils of Epirus, as a function of rainfall class. Values of means are expressed in mg/kg.

Table S11 presents the results of the statistical tests for the two classes of mean rainfall. Using the same reasoning as with altitude, we set the limit at 70 mm. In contrast to altitude, the average amount of rainfall does affect the concentration of some phenolic constituents and the total phenolics content. The two classes exhibited a highly statistically significant difference for total phenolics, (**3**), and (**4**) (*p* < 0.001), as well as for (**7**) and (**8**) (*p* < 0.05). However, there was no significant difference between the two classes for (**5**) and (**6**). The averages of the biophenols, (**3**) and (**4**), and the total phenolics were higher in class 2, which included olive oil samples originating from areas with an average rainfall of more than 70 mm. Class 1 olive oil samples had higher levels of (**7**) and (**8**) compared with those in class 2, while the averages of (**5**) and (**6**) were nearly identical (see Figure 9).

All the samples from the Arta region, characterized by high levels of oleokoronal (**7**) and oleomissional (**8**), are in class 1, whereas the majority of samples from Preveza, known for high total phenolics content, fall into class 2 (Table S12). These findings align with our earlier results but do not definitively clarify whether it is the region or the variety that primarily influences phenolic content. However, the same pattern does not hold for the samples from Thesprotia. A crosstab in Table S12 reveals that most of its samples belong to class 2. This would lead us to expect that the components prevalent in these samples, (**5**) and (**6**), would also be more abundant in this class, as seen in the other two prefectures. Surprisingly, this is not the case; the concentrations of these aglycone compounds not only lack significant differences but also have similar mean values. In fact, for (**6**), the average is exactly the same, at 18 mg/kg (Table S11). In the other crosstab table (variety–rainfall class, Table S13), we find that the local variety of the Thesprotia region, Mesokarpos, is equally present in both classes, with seven samples in each class. This particular cultivar is known to be rich in (**5**) but primarily in (**6**). This explains the average results for these aglycone components, underscoring both the strong influence of olive variety on phenolic content and the secondary role of the region factor in phenolic concentrations in the studied samples.

The last climatological factor examined was the average temperature. Class 1 of the dichotomous variable contained samples with an average temperature of the region of origin below 16.5 °C, while the other class had an average temperature equal to or greater than 16.5 °C. Table S14 reveals that the two categories do not exhibit significant differences in terms of average phenolic content. This suggests that the temperature factor in the Epirus regions does not significantly affect phenolic content.

### 2.7. Phenolics Amounts Correlation

Correlation analysis was conducted using the non-parametric Spearman coefficient, which assesses the strength and direction of the relationship between two quantitative variables. This coefficient can range from −1 to +1, with the sign indicating the direction of the correlation and the absolute value denoting its strength. Additional information is available in the Supplementary Materials (Table S15) [61]. The correlation analysis involved the relationship between the total phenolics and the sums of the D1, D2, and D3 indexes.

**Figure 10:**
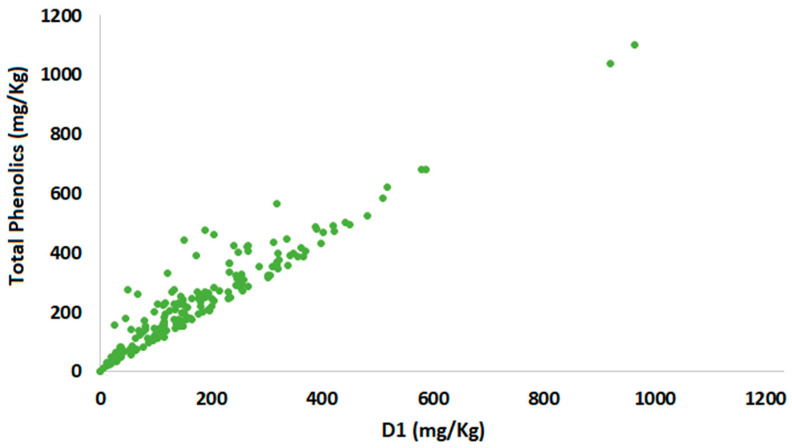
Total phenolics scatter plot versus index D1.

For all pairs of variables, a positive correlation was observed, indicating that the total phenolic content is proportional to the individual phenolic components. According to Table S15, the correlation between the total phenolic content and the sum of oleocanthal (**3**) and oleacein (**4**) (index D1) is very strong, while the effect of the sum of oleokoronal (**7**) and oleomissional (**8**) (index D3) is moderately strong (Table 6). The correlation between total phenolics and D1 can be characterized as linear (R^2^ = 0.85), while for the other two pairs, the R^2^ values were calculated as 0.51 and 0.21, indicating a nonlinear correlation. In addition to the R^2^ values, the scatter plots illustrate the type of correlation (linear or nonlinear), as shown in Figure 10, Figures S4 and S5.

## 3. Materials and Methods

### 3.1. Chemicals and Standards

Syringaldehyde (>98% purity), used as internal standard (IS), was purchased from Thermal Scientific (Odessa, TX, USA). IS solution was prepared in CDCl_3_ at a concentration of 0.025 mg/mL, kept in refrigerator, and allowed to come to room temperature before use. Deuterated chloroform (CDCl_3_) was obtained from Deutero (Kastellaun, Germany) and used without further purification.

### 3.2. Chemicals and Standards

**Figure 11:**
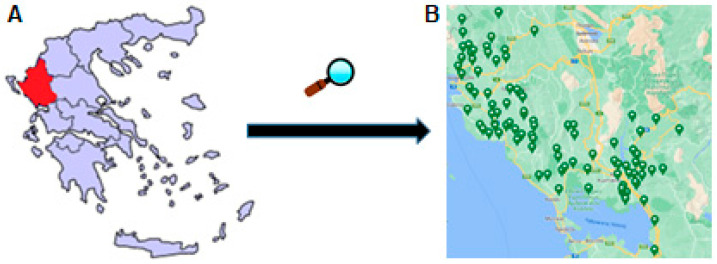
(**A**) Map of Greece with the Epirus region indicated with red; (**Β**) Samples collected from the Epirus area are marked with map markers.

The samples used in the study were obtained from olives (*Olea europaea* L.) harvested and extracted in the time period from October 2022 to March 2023. In total, 180 samples were collected from the Epirus region, 5 from Messinia, 6 from Zakynthos, 7 from Corfu, and 2 from Crete. These samples encompassed seven different olive varieties and were sourced from small-scale producers, in collaboration with the olive mills in the aforementioned regions, which could verify their monovarietal origin. For further details, please refer to Table 7, Table S9 and Figure 11.

### 3.3. Proton Nuclear Magnetic Resonance (^1^H NMR) Spectroscopic Analyses

#### 3.3.1. General

^1^H NMR spectra were acquired using a Bruker Avance NEO spectrometer (Bruker, Karlsruhe, Germany) operating at 500.13 MHz and equipped with a cryoprobe. For each sample, 200 μL (150–180 mg) of olive oil was mixed with 400 μL of CDCl_3_ in a 5 mm diameter NMR tube. The mixture contained 0.05% tetramethylsilane (TMS) as a reference compound and 0.01 mg of syringaldehyde. CDCl_3_ was used as solvent because it does not react with the studied phenolic compounds, unlike other solvents, such as methanol and water [62]. Syringaldehyde was selected as the internal standard as proposed by Magiatis et al. Briefly, none of the studied samples contained syringaldehyde or any other peaks that would overlap with its aldehydic proton. Additionally, syringaldehyde is a cheap, stable, and well-soluble compound in CDCl_3_, and its ^1^H NMR spectrum is very simple, minimizing the possibility of overlap with other interesting spectrum regions [46]. Each sample was recorded in two different ^1^H NMR experiments (1D ^1^H NMR and multi-suppression) and was processed using Topspin 4.1.1 (Bruker Analytik GmbH, Rheinstetten, Germany). Deconvolution was performed, also via Topspin 4.1.1, automatically (dcon). The spectra were recorded at 298 K. Automated tuning, matching, and shimming were used.

#### 3.3.2. 1D ^1^H NMR

1D ^1^H NMR spectra (standard experiment, SE) were collected using a 30° flip angle and a spectral width of 20 ppm, using the same receiver gain value (automatically determined and set to the value of 10), using a relaxation delay of 5 s. The number of transients was 8 and the acquisition time was 3.28 s. A total of 64 K data points were collected, and the FIDs were treated using a line-broadening exponential function of 0.3 Hz. Phase adjustment and baseline correction were performed manually using Topspin version 4.07.

#### 3.3.3. Multi-Suppression Experiment (MSE)

Experiments were performed using the build in noesygppr1d with wvm for suppression peak list. A 90° flip angle and a spectral width of 20 ppm were used. The relaxation delay was set to 3 s. Prior to the measurement, each sample was equilibrated (4 dummy scans). The number of transients was 256 and the acquisition time was 3.28 s. A total of 64 K data points were collected using the same receiver gain value (automatically determined and set to the value of 20.2). Additionally, the mixing time was set to 10 ms and the width of narrow, off-resonance suppression was set to 4 Hz. The amplitude- and phase-modulated pulse was applied in a list of frequencies as provided in Table S16. More specifically, 17 frequencies that correspond to the major peaks of EVOO were selected. The dd signals at 4.301 and 4.141 ppm were intentionally not selected for suppression in order to facilitate a comparison of peak intensities between the two experiments (SE and MSE). Consequently, the RG increased to 20.2 and the signal-to-noise ratio was found to be more than 37 times greater compared with the SE.

### 3.4. Determination of Phenolic Compounds

Quantification of total phenolic constituents was performed using Equation (1) as previously described [63]:

(1) $$mx=\frac{Ix}{Istd}\times \frac{Nstd}{Nx}\times \frac{Mx}{Mstd}\times mstd\times Pstd$$

where m_x_ is the determined weight of each compound; I_x_ and I_std_ are the integrated signal areas of the analyte x and the internal standard (std), respectively; N_x_ and N_std_ are the numbers of resonating nuclei; M_x_ and M_std_ are the molar masses; m_std_ is the weighed mass of the internal standard (0.01 mg); and P_std_ is the purity of the internal standard. P_std_ was set to 0.985, as was the purity of the internal standard.

### 3.5. Statistical Analysis

The chemical profiles of the examined olive oil samples were assessed in terms of variety, harvest time (month), and geographic origin (region and municipality). To assess the significance of the differences between these factors, we applied the non-parametric Mann–Whitney U test to the data for variables with two categories and the Kruskal–Wallis test for variables with more than two categories, as the distribution of all phenolic content was non-normal (as shown in Tables S17–S54). All analyses were performed at a significance level of *p* = 0.05, using SPSS v.28.0.1.0 software for Windows (IBM SPSS Statistics 2023, IBM Corp., Armonk, NY, USA).

## 4. Conclusions

This study involved the direct identification and quantification of phenolic compounds in Epirus olive oils, known for their valuable health properties, using a multiple suppression NMR experiment. Among the Epirus region samples, those from Thesprotia and Preveza recorded the highest concentrations of the polyphenols under investigation, with a mean of 246.02 and a median of 224.51—values very close to the limits set by the EU for health claims. High total phenolic values were found in the samples from Zakynthos, among those from other regions. It is important to note that the last conclusion is made with some reservation, as the selection of olive mills outside Epirus was performed randomly. The same Epirus samples also exhibited high values for the polyphenols oleocanthal (**3**) and oleacein (**4**), indicating a strong correlation with the total phenolic compounds. This correlation was further confirmed using the Spearman correlation coefficient (r = 0.924), indicating a very strong relationship. As for other phenolic components, the sum of the under-studied aglycone compounds (D2) was found to be highest in a sample from Thesprotia, while a sample from Arta had the highest value for the sum of D3 (oleokoronal (**7**) + oleomissional (**8**)).

The non-parametric tests revealed a significant effect of olive variety and harvest month on the phenolic content of the samples. Kalamon exhibited a higher mean for total phenolics and (**3**), while the Lianolia cultivar showed higher D levels of (**4**). The local variety of Thesprotia, Mesokarpos, had elevated levels of ligstroside aglycone (**5**) and oleuropein aglycone (**6**), and the Koroneiki variety had higher levels of (**7**) and (**8**). Regarding the harvest month, our results are in line with the existing literature, as olive oils extracted from unripe olives tend to have a higher polyphenol content. Finally, the Mann–Whitney U test for the variables related to the climatic factors of the region of origin showed a statistically significant difference between the categories of mean precipitation but not for those of mean temperature and altitude. This suggests that region has a weaker effect on polyphenol content compared with cultivar and harvest month.

Overall, Epirus olive oils hold significant promise in terms of their biophenolic content and have the potential to be categorized as high-phenolic olive oils with optimization in olive tree maintenance, fruit collection, and olive oil extraction methods.

## Figures and Tables

**Table 1:** Variety and region of olive oil samples with concentrations of total phenolics above 500 mg/kg.

Variety	Region	Total Phenolics (mg/kg)
Kalamon	Thesprotia	1100
Kalamon	Thesprotia	1039
Lianolia	Thesprotia	683
Lianolia	Preveza	680
Lianolia	Preveza	622
Lianolia	Preveza	586
Koroneiki	Preveza	567
Lianolia	Thesprotia	525
Koroneiki	Zakynthos	520
Koroneiki	Zakynthos	514
Lianolia	Preveza	502

**Table 2:** Variety and region of the 10 top olive oil samples in sum oleocanthal (**3**) and oleacein (**4**) (D1).

Variety	Region	Oleocanthal (3) (mg/kg)	Oleacein (4) (mg/kg)	D1 (mg/kg)
Kalamon	Thesprotia	863 ^a^	101	964
Kalamon	Thesprotia	712	208	920
Lianolia	Thesprotia	344	243	587
Lianolia	Preveza	291	288 ^b^	579
Lianolia	Preveza	303	216	519
Lianolia	Preveza	318	192	510
Lianolia	Thesprotia	320	161	481
Amfissis	Arta	393	57	450
Lianolia	Preveza	273	169	442
Koroneiki	Arta	241	182	423

^a^ = highest sample with (**3**); ^b^ = highest sample with (**4**).

**Table 3:** Kruskal–Wallis test for total phenolics, oleocanthal (**3**), and oleacein (**4**) among olive varieties, with average (Avg.) and median (Med.) values expressed in mg/kg.

	Total Phenolics				Oleocanthal (3)				Oleacein (4)			
Variety	N	Avg.	Med.		N	Avg.	Med.		N	Avg.	Med.	
Amfissis	34	188 ± 21	164	Kruskal–Wallis 18.70 df 6 sig 0.005	34	93 ± 12	89	Kruskal–Wallis 28.88 df 6 sig <0.001	34	39 ± 7	28	Kruskal–Wallis 27.64 df 6 sig <0.001
Koroneiki	26	249 ± 29	229		26	92 ± 11	87		26	68 ± 10	54	
Lianolia	63	297 ± 20	287		63	160 ± 11	153		63	88 ± 8	78	
Mesokarpos	14	243 ± 33	254		14	111 ± 21	98		14	53 ± 12	38	
Table olives	27	182 ± 23	154		27	75 ± 9	65		27	42 ± 7	40	
Kalamon	10	321 ± 128	163		10	221 ± 98	77		10	52 ± 20	38	
Agria	4	162 ± 29	187		4	93 ± 15	101		4	31 ± 8	35	

Avg. = Average; Med. = Median; df = degree of freedom; sig = significance.

**Table 4:** Kruskal–Wallis test for total phenolics, oleocanthal (**3**), and oleacein (**4**) according to the month of olive oil production, with average (Avg.) and median (Med.) values expressed in mg/kg.

	Total Phenolics				Oleocanthal (3)				Oleacein (4)			
Harvest Month	N	Avg.	Med.		N	Avg.	Med.		N	Avg.	Med.	
October	20	285 ± 51	238	Kruskal–Wallis 42.90 df = 3 sig < 0.001	20	160 ± 41	106	Kruskal–Wallis 52.85 df = 3 sig < 0.001	20	61 ± 12	51	Kruskal–Wallis 39.56 df = 3 sig < 0.001
November	99	297 ± 16	276		99	149 ± 10	136		99	81 ± 5	74	
December	41	162 ± 19	142		41	77 ± 9	64		41	36 ± 7	20	
January to March	18	116 ± 26	72		18	36 ± 3	22		18	27 ± 9	11	

Avg. = Average; Med. = Median; df = degree of freedom; sig = significance.

**Table 5:** Kruskal–Wallis test for total phenolics, oleocanthal (**3**), and oleacein (**4**) among Epirus prefectures, with average (Avg.) and median (Med.) values expressed in mg/kg.

	Total Phenolics				Oleocanthal (3)				Oleacein (4)			
Prefecture	N	Avg.	Med.		N	Avg.	Med.		N	Avg.	Med.	
Arta	62	185 ± 14	170	Kruskal–Wallis 12.48 df = 2 sig = 0.002	62	81 ± 8	72	Kruskal–Wallis 20.27 df = 2 sig < 0.001	62	43 ± 5	37	Kruskal–Wallis 14.86 df = 2 sig < 0.001
Preveza	52	269 ± 22	251		52	136 ± 11	122		52	81 ± 8	72	
Thesprotia	63	287 ± 25	261		63	151 ± 18	119		63	67 ± 7	45	

Avg. = Average; Med. = Median; df = degree of freedom; sig = significance.

**Table 6:** Spearman’s rho values.

Variables	Correlation Coefficient	Sig. (2-Tailed)
Total phenolics—D1	+0.924	<0.001
Total phenolics—D2	+0.717	<0.001
Total phenolics—D3	+0.462	<0.001

**Table 7:** Origins, cultivars, and number of olive oil samples.

	Epirus					Ionian Islands		
Variety	Arta	Preveza	Thesprotia	Messinia	Crete	Corfu	Zakynthos	Total Number
Amfissis	31	2	1	-	-	-	-	34
Koroneiki	10	9	7	3	2	5	3	39
Lianolia	2	41	20	4	-	-	3	70
Mesokarpos	-	-	14	-	-	-	-	14
Table olives	18	-	9	-	-	-	-	27
Kalamon	1	1	8	-	-	-	-	10
Agria	-	-	4	-	-	-	-	4
Total number	62	53	63	7	2	5	6	198

## Data Availability

Data are contained within the article and Supplementary Materials.

## Supplementary Materials

The following supporting information can be downloaded at: https://www.mdpi.com/article/10.3390/molecules29051111/s1, Table S1: NMR chemical shifts of protons of some compounds present in EVOO or generated during processing and storage; Table S2: Variety and region of the 10 top olive oil samples in sum ligstroside aglycone (**5**) and oleuropein aglycone (**6**) (D2); Table S3: Variety and region of the 10 top olive oil samples in sum oleokoronal (**7**) and oleomissional (**8**) (D3); Table S4: Amounts of phenolics studied in the present study; Table S5: Test of Kruskal–Wallis H, average (Avg.) and median (Med.) of ligstroside aglycone (**5**) and oleuropein aglycone (**6**) in relation to the olive variety. Values of Avg. and Med. are expressed in mg/kg; Table S6: Test of Kruskal–Wallis H, average (Avg.) and median (Med.) of oleokoronal (**7**) and oleomissional (**8**) in relation to the olive variety. Values of Avg. and Med. are expressed in mg/kg; Table S7: Test of Kruskal–Wallis H, average (Avg.) and median (Med.) of ligstroside aglycone (**5**), according to the month of olive oil production. Values of Avg. and Med. are expressed in mg/kg; Table S8: Test of Kruskal–Wallis H, average (Avg.) and median (Med.) of ligstroside aglycone (**5**), oleuropein aglycone (**6**), and oleokoronal (**7**) among prefectures of Epirus. Values of Avg. and Med. are expressed in mg/kg; Table S9: Origins, altitude, month of harvest, and analysis and variety of olive oil samples; Table S10: Results of Mann–Whitney U test between the two classes of altitude; Table S11: Results of Mann–Whitney U test and average (Avg.) of total phenolics, oleocanthal (**3**), oleacein (**4**), ligstroside aglycone (**5**), oleuropein aglycone (**6**), oleokoronal (**7**), and oleomissional (**8**) according to the two classes of average rainfall. Values of Avg. are expressed in mg/kg; Table S12: Crosstab table of rainfall class—prefecture; Table S13: Crosstab table of rainfall class—variety; Table S14: Results of Mann–Whitney U test between the two classes of temperature mean; Table S15: Interpretation of the correlation coefficient *r*; Table S16: The frequencies that were selected for suppression in the MSE method, by ppm; Table S17: Normality tests of mean amounts of total phenolics among the olive varieties; Table S18: Normality tests of mean amounts of oleocanthal (**3**) among the olive varieties; Table S19: Normality tests of mean amounts of oleacein (**4**) among the olive varieties; Table S20: Normality tests of mean amounts of ligstroside aglycone (**5**) among the olive varieties; Table S21: Normality tests of mean amounts of oleuropein aglycone (**6**) among the olive varieties; Table S22: Normality tests of mean amounts of oleokoronal (**7**) among the olive varieties; Table S23: Normality tests of mean amounts of oleomissional (**8**) among the olive varieties; Table S24: Normality tests of mean amounts of total phenolics according to the harvest month; Table S25: Normality tests of mean amounts of oleocanthal (**3**) according to the harvest month; Table S26: Normality tests of mean amounts of oleacein (**4**) according to the harvest month; Table S27: Normality tests of mean amount of ligstroside aglycone (**5**) according to the harvest month; Table S28: Normality tests of mean amounts of total phenolics by prefecture; Table S29: Normality tests of mean amounts of oleocanthal (**3**) by prefecture; Table S30: Normality tests of mean amounts of oleacein (**4**) by prefecture; Table S31: Normality tests of mean amounts of ligstroside aglycone (**5**) by prefecture; Table S32: Normality tests of mean amounts of oleuropein aglycone (**6**) by prefecture; Table S33: Normality tests of mean amounts of oleokoronal (**7**) by prefecture; Table S34: Normality tests of mean amounts of total phenolics by altitude class; Table S35: Normality tests of mean amounts of oleocanthal (**3**) by altitude class; Table S36: Normality tests of mean amounts of oleacein (**4**) by altitude class; Table S37: Normality tests of mean amounts of ligstroside aglycone (**5**) by altitude class; Table S38: Normality tests of mean amounts of oleuropein aglycone (**6**) by altitude class; Table S39: Normality tests of mean amounts of oleokoronal (**7**) by altitude class; Table S40: Normality tests of mean amounts of oleomissional (**8**) by altitude class; Table S41: Normality tests of mean amounts of total phenolics by rainfall class; Table S42: Normality tests of mean amounts of oleocanthal (**3**) by rainfall class; Table S43: Normality tests of mean amounts of oleacein (**4**) by rainfall class; Table S44: Normality tests of mean amounts of ligstroside aglycone (**5**) by rainfall class; Table S45: Normality tests of mean amounts of oleuropein aglycone (**6**) by rainfall class; Table S46: Normality tests of mean amounts of oleokoronal (**7**) by rainfall class; Table S47: Normality tests of mean amounts of oleomissional (**8**) by rainfall class; Table S48: Normality tests of mean amounts of total phenolics by temperature class; Table S49: Normality tests of mean amounts of oleocanthal (**3**) by temperature class; Table S50: Normality tests of mean amounts of oleacein (**4**) by temperature class; Table S51: Normality tests of mean amounts of ligstroside aglycone (**5**) by temperature class; Table S52: Normality tests of mean amounts of oleuropein aglycone (**6**) by temperature class; Table S53: Normality tests of mean amounts of oleokoronal (**7**) by temperature class; Table S54: Normality tests of mean amounts of oleomissional (**8**) by temperature class; Figure S1: Pairwise comparisons of variety. (A) Average of ligstroside aglycone (**5**); (B) Average of oleuropein aglycone (**6**); (C) Average of oleokoronal (**7**); (D) Average of oleomissional (**8**). Significance values have been adjusted by the Bonferroni correction for multiple tests; Figure S2: Pairwise comparisons of harvest month of average of ligstroside aglycone (**5**). Significance values have been adjusted by the Bonferroni correction for multiple tests; Figure S3: Pairwise comparisons of prefecture. (A) Average of total phenolics; (B) Average of oleocanthal (**3**); (C) Average of oleacein (**4**); (D) Average of ligstroside aglycone (**5**); (E) Average of oleuropein aglycone (**6**); (F) Average of oleokoronal (**7**). Significance values have been adjusted by the Bonferroni correction for multiple tests; Figure S4: Total phenolics scatter plot versus index D2; Figure S5: Total phenolics scatter plot versus index D3.
