# Supplementary material for: NMR Analysis of Extra Virgin Olive Oil of the Epirus Region of Greece with Emphasis on Selected Phenolic Compounds
Source: Molecules. 2024 Mar 1;29(5):1111. doi: 10.3390/molecules29051111 (PMC10934859; doi:10.3390/molecules29051111)
Supplement: Supplementary file 1 [file molecules-29-01111-s001.zip › molecules-2894169-supplementary.pdf]

NMR analysis of extra virgin olive oil of the Epirus region of Greece with emphasis on selected phenolic compounds.

Theodoros Tsolis [1,*], Dimitra Kyriakou [1], Evangelia Sifnaiou [1], Dimitrios Thomos [1], Dimitrios Glykos [1], Constantinos G Tsiafoulis [1,2] and Achilleas Garoufis [1,3,*]

[1] Laboratory of Inorganic Chemistry, Department of Chemistry, University of Ioannina, 45110 Ioannina, Greece; t.tsolis@uoi.gr (T.T.); d.kyriakou@uoi.gr (D.K.); e.sifnaiou@uoi.gr (E.S.); d.thomos@uoi.gr (D.T.); d.glykos@uoi.gr (D.G.); ctsiafou@uoi.gr (C.T.)
[2] School of Science & Technology, Hellenic Open University, Patras, Greece
[3] Institute of Materials Science and Computing, University Research Centre of Ioannina (URCI), 45110 Ioannina, Greece
* Correspondence: t.tsolis@uoi.gr; agaroufi@uoi.gr

Table of Contents

Table S1: NMR chemical shifts of protons of some compounds present in EVOO or generated during processing and storage 4

Table S2. Variety and Region of the 10 top olive oil samples in sum ligstroside aglycone (**5**) and oleuropein aglycone (**6**) (D2)........ 4

Table S3. Variety and Region of the 10 top olive oil samples in sum oleokoronal (**7**) and oleomissional (**8**) (D3) 5

Table S4. Amounts of phenolics studied in the present study........ 5

Table S5. Test of Kruskal Wallis H, Average (Ave) and Median (Med) of ligstroside aglycone (**5**) and oleuropein aglycone (**6**) in relation to the olive variety. Values of Ave and Med are expressed in mg/Kg........ 10

Table S6. Test of Kruskal Wallis H, Average (Ave) and Median (Med) of oleokoronal (**7**) and oleomissional (**8**) in relation to the olive variety. Values of Ave and Med are expressed in mg/Kg. 10

Figure S1. Pairwise Comparisons of Variety. A) Average of ligstroside aglycone (**5**); B) Average of oleuropein aglycone (**6**); C) Average of oleokoronal (**7**); D) Average of oleomissional (**8**). Significance values have been adjusted by the Bonferroni correction for multiple tests. 11

Table S7. Test of Kruskal Wallis H, Average (Ave) and Median (Med) of ligstroside aglycone (**5**), among the month of olive oil production. Values of Ave and Med are expressed in mg/Kg. 11

Figure S2. Pairwise Comparisons of harvest month of average of ligstroside aglycone (**5**). Significance values have been adjusted by the Bonferroni correction for multiple tests. 12

Table S8. Test of Kruskal Wallis H, Average (Ave) and Median (Med) of ligstroside aglycone (**5**), oleuropein aglycone (**6**) and oleokoronal (**7**) among prefectures of Epirus. Values of Ave and Med are expressed in mg/Kg........ 12

Table S9: Origins, altitude, month of harvest and analysis and variety of olive oil samples. 13

Table S11. Results of Mann-Whitney U test and Average (Ave) of total phenolics, oleocanthal (**3**), oleocein (**4**), ligstroside aglycone (**5**), oleuropein aglycone (**6**), oleokoronal (**7**) and oleomissional (**8**) among the two classes of average rainfall. Values of Ave are expressed in mg/Kg. 17

Table S12. Crosstab table of rainfall class – prefecture........ 18

Table S13. Crosstab table of rainfall class – variety. 18

Table S14. Results of Mann-Whitney U test between the two classes of temperature mean. 18

Table S15. Interpretation of the correlation coefficient *r*... 18

Figure S4. Total phenolics scatter plot versus index D2. 19

Figure S5. Total phenolics scatter plot versus index D3. 19

Table S16: The frequencies that were selected for suppression in the MSE method, at ppm. 20

Table S17. Normality tests of mean amount of total phenolics among the olive variety. 20

Table S18. Normality tests of mean amount of oleocanthal (**3**) among the olive variety. 20

Table S19. Normality tests of mean amount of oleocein (**4**) among the olive variety. 21

Table S20. Normality tests of mean amount of ligstroside aglycone (**5**) among the olive variety. 21

Table S21. Normality tests of mean amount of oleuropein aglycone (**6**) among the olive variety.................................. 21

Table S22. Normality tests of mean amount of oleokoronal (**7**) among the olive variety. 22

Table S23. Normality tests of mean amount of oleomissional (**8**) among the olive variety. 22

Table S24. Normality tests of mean amount of total phenolics among the harvest month. 22

Table S25. Normality tests of mean amount of oleocanthal (**3**) among the harvest month. 22

Table S26. Normality tests of mean amount of oleocein (**4**) among the harvest month. 23

Table S27. Normality tests of mean amount of ligstroside aglycone (**5**) among the harvest month. 23

Table S28. Normality tests of mean amount of total phenolics among the prefecture. 23

Table S29. Normality tests of mean amount of oleocanthal (**3**) among the prefecture. 23

Table S30. Normality tests of mean amount of oleocein (**4**) among the prefecture. 23

Table S31. Normality tests of mean amount of ligstroside aglycone (**5**) among the prefecture. 24

Table S32. Normality tests of mean amount of oleuropein aglycone (**6**) among the prefecture...................................... 24

Table S33. Normality tests of mean amount of oleokoronal (**7**) among the prefecture. 24

Table S34. Normality tests of mean amount of total phenolics among the altitude class. 24

Table S35. Normality tests of mean amount of oleocanthal (**3**) among the altitude class. 24

Table S36. Normality tests of mean amount of oleocein (**4**) among the altitude class. 25

Table S37. Normality tests of mean amount of ligstroside aglycone (**5**) among the altitude class.. 25

Table S38. Normality tests of mean amount of oleuropein aglycone (**6**) among the altitude class................................. 25

Table S39. Normality tests of mean amount of oleokoronal (**7**) among the altitude class. 25

Table S40. Normality tests of mean amount of oleomissional (**8**) among the altitude class. 25

Table S41. Normality tests of mean amount of total phenolics among the rainfall class. 25

Table S42. Normality tests of mean amount of oleocanthal (**3**) among the rainfall class. 26

Table S43. Normality tests of mean amount of oleocein (**4**) among the rainfall class. 26

Table S44. Normality tests of mean amount of ligstroside aglycone (**5**) among the rainfall class. 26

Table S45. Normality tests of mean amount of oleuropein aglycone (**6**) among the rainfall class.................................. 26

Table S46. Normality tests of mean amount of oleokoronal (**7**) among the rainfall class. 26

Table S47. Normality tests of mean amount of oleomissional (**8**) among the rainfall class. 26

Table S48. Normality tests of mean amount of total phenolics among the temperature class. 27

Table S49. Normality tests of mean amount of oleocanthal (**3**) among the temperature class. 27

Table S50. Normality tests of mean amount of oleocein (**4**) among the temperature class. 27

Table S51. Normality tests of mean amount of ligstroside aglycone (**5**) among the temperature class. 27

Table S52. Normality tests of mean amount of oleuropein aglycone (**6**) among the temperature class. 27

Table S53. Normality tests of mean amount of oleokoronal (**7**) among the temperature class. 27

Table S54. Normality tests of mean amount of oleomissional (**8**) among the temperature class. 28

Table S1: NMR chemical shifts of protons of some compounds present in EVOO or generated during processing and storage [1,2].

Major compounds				Minor compounds			
Peak	Chemical shift (ppm)	Multiplicity	Functional Group	Peak	Chemical shift (ppm)	Multiplicity (J in Hz)	Compound Functional Group
A	0.88	t	-(CH$_3$)	3	9.23	d (J = 2.0)	-**CHO** (C-1)
	0.89	t			9.62	os	-**CHO** (C-3)
B	1.22-1.42	m	-(CH$_2$)$_n$-	4	9.21	d (J = 2.0)	-**CHO** (C-1)
C	1.52-1.70	m	-(CH$_2$)$_n$-		9.63	os	-**CHO** (C-3)
D	1.94-2.14	m	-OCO-CH$_2$-**CH$_2$**-	5	9.506	d (J = 1.7)	-**CHO** (C-1)
E	2.31	dt	-OCO-**CH$_2$**-CH$_2$-	6	9.515	d (J = 1.7)	-**CHO** (C-1)
F	2.77-2.80	t	=CH-**CH$_2$**-CH=	7	9.22	os	-**CHO** (C-1)
G	4.12	dd	-**CH$_2$**-OCO-R		11.75	d (J = 12.7)	=CH-**OH** (C-3)
	4.30	dd		5S,4R-7a	9.22	os	**CHO** (C-1)
H	5.27	m	-**CH**-OCO-R		9.68	d (J = 2.7)	**CHO** (C-3)
I	5.30-5.47	m	-**CH=CH**-	5S,4S-7a	9.22	os	**CHO** (C-1)
Secondary oxidation compounds					9.453	d (J = 2.7)	**CHO** (C-3)
a	9.502	d (J = 7.9)	(E)-2-alkenals				
b	9.498	d (J = 7.9)	(E)-2-alkenals	8	9.20	os	-**CHO** (C-1)
**c**	9.750	t	Alkanals		11.77	d (J = 12.7)	=CH-**OH** (C-3)
				5S,4R-8a	9.19	os	**CHO** (C-1)
					9.67	d (J = 2.7)	**CHO** (C-3)
				5S,4S-8a	9.19	os	**CHO** (C-1)
					9.448	d (J = 2.7)	**CHO** (C-3)

Table S2. Variety and Region of the 10 top olive oil samples in sum ligstroside aglycone (**5**) and oleuropein aglycone (**6**) (D2).

Variety	Region	ligstroside aglycone (5) (mg/Kg)	oleuropein aglycone (6) (mg/Kg)	D2 (mg/Kg)
Mesokarpos	Thesprotia	22	134[b]	156
Table olives	Thesprotia	64	77	141
Koroneiki	Zakynthos	80[a]	34	114
Koroneiki	Thesprotia	26	85	111
Koroneiki	Thesprotia	41	64	105
Koroneiki	Zakynthos	46	58	104
Koroneiki	Corfu	66	36	102
Koroneiki	Zakynthos	50	51	101
Mesokarpos	Thesprotia	31	69	100

Table olives	Thesprotia	24	74	98

a = highest sample with ligstroside aglycone (**5**); b = highest sample with oleuropein aglycone (**6**)

Table S3. Variety and Region of the 10 top olive oil samples in sum oleokoronal (**7**) and oleomissional (**8**) (D3).

Variety	Region	oleokoronal (7) (mg/Kg)	oleomissional (8) (mg/Kg)	D3 (mg/Kg)
Amfissis	Arta	125	61	187
Koroneiki	Crete	112	51	163
Koroneiki	Preveza	108	51	159
Table olives	Arta	114	38	152
Table olives	Thesprotia	130[a]	22	152
Mesokarpos	Thesprotia	54	79[b]	133
Mesokarpos	Thesprotia	97	31	128
Koroneiki	Thesprotia	88	37	125
Lianolia	Preveza	71	49	120
Lianolia	Thesprotia	86	18[b]	104

a = highest sample with oleokoronal (**7**); b = highest sample with oleomissional (**8**)

Table S4. Amounts of phenolics studied in the present study

Sample	Geographical factor		Variety	Amounts of phenolics (mg/Kg)						
	Town	Province		Total	oleocanthal (3)	oleocein (4)	ligstroside aglycone (5)	oleuropein aglycone (6)	oleokoronal (7)	Oleomissional (8)
1	Foteino	Arta	Amfissis	220.27	59.03	142.03	12.52	0.00	6.69	0.00
2	Petra	Arta	Amfissis	83.34	77.59	0.00	5.76	0.00	0.00	0.00
3	Glikorizo	Arta	Amfissis	496.46	393.15	57.12	46.20	0.00	0.00	0.00
4	Neoxwri	Arta	Amfissis	270.44	180.52	50.13	19.73	4.12	13.19	2.75
5	Menidi	Arta	Koroneiki	471.83	240.91	181.77	11.28	17.13	16.45	4.29
6	Megarxi	Arta	Amfissis	105.52	66.71	27.90	5.43	1.13	4.35	0.00
7	Foteino	Arta	Amfissis	277.15	93.07	40.12	27.36	27.48	78.10	11.02
8	Dimario	Arta	Amfissis	267.65	122.83	66.52	15.85	15.45	38.15	8.85
9	Kompoti	Arta	Amfissis	74.54	55.61	2.83	5.33	2.22	8.54	0.00
10	Kleisto	Arta	Amfissis	365.47	126.97	105.61	23.19	33.03	63.43	13.25
11	Neoxwraki	Arta	Amfissis	210.80	113.88	20.57	19.17	8.43	44.52	4.23
12	Foteino	Arta	Amfissis	171.16	120.38	27.47	8.00	7.31	8.02	0.00
13	Menidi	Arta	Amfissis	328.51	123.14	131.33	13.56	23.26	29.13	8.11
14	Petra	Arta	Amfissis	158.76	99.06	15.41	5.11	30.97	8.20	0.00
15	Anoixiatiko	Arta	Amfissis	169.50	114.41	0.00	20.74	1.08	33.28	0.00
16	Menidi	Arta	Amfissis	140.42	81.46	24.63	8.23	4.30	18.57	3.23
17	Foteino	Arta	Amfissis	271.30	138.71	76.60	10.17	10.62	22.43	12.77
18	Kleidi	Arta	Amfissis	363.43	141.14	90.59	18.39	22.41	73.78	17.12
19	Gorgomilos	Preveza	Koroneiki	437.08	175.10	136.91	25.74	26.88	49.64	22.81
20	Loutropoulo	Arta	Koroneiki	75.05	65.91	0.00	5.22	0.00	3.92	0.00

21	Petra	Arta	Amfissis	241.79	102.45	46.73	8.11	10.59	56.93	16.98
22	Makriniada	Arta	Amfissis	111.80	90.49	12.35	4.97	0.00	3.99	0.00
23	Paliokatouno	Arta	Amfissis	462.01	101.95	102.12	24.41	46.90	125.31	61.33
24	Petra	Arta	Amfissis	282.93	139.28	64.80	10.96	6.68	43.98	17.22
25	Neoxwraki	Arta	Amfissis	130.57	75.48	32.76	4.62	2.89	14.82	0.00
26	Loutrotopos	Arta	Amfissis	57.17	53.48	0.84	2.84	0.00	0.00	0.00
27	Paxikalamos	Arta	Koroneiki	200.61	59.21	37.25	14.55	8.44	77.79	3.38
28	Kompoti	Arta	Amfissis	35.04	28.82	0.80	1.80	0.00	3.61	0.00
29	Katw Athnamanio	Arta	Koroneiki	324.48	130.89	113.69	17.39	22.70	26.16	13.66
30	Megarxi	Arta	Amfissis	204.79	88.07	37.50	14.37	13.34	44.83	6.69
31	Parga	Preveza	Koroneiki	566.62	180.58	137.60	33.50	55.98	107.51	51.45
32	Tzara	Thesprotia	Lianolia	264.76	140.57	54.28	15.10	17.52	30.28	7.03
33	Faskomilia	Thesprotia	Koroneiki	184.94	109.62	47.95	10.14	7.06	10.17	0.00
34	Eleutheri	Thesprotia	Lianolia	387.37	236.68	128.98	13.30	1.74	6.67	0.00
35	Margariti	Thesprotia	Mesokarpos	72.98	58.81	3.82	6.90	0.00	3.46	0.00
36	Katavothra	Thesprotia	Lianolia	425.39	212.19	53.18	25.71	30.43	85.94	17.95
37	Themelo	Thesprotia	Lianolia	355.70	212.23	73.49	18.06	18.86	29.63	3.44
38	Ag. Kuriaki	Preveza	Koroneiki	253.48	87.86	91.05	12.04	16.76	28.97	16.80
39	Morfi	Thesprotia	Table olives	406.93	225.75	144.22	12.64	8.80	8.45	7.06
40	Eleutheri	Thesprotia	Lianolia	433.24	253.22	144.14	16.11	12.01	6.15	1.61
41	Palaiokastro	Thesprotia	Mesokarpos	356.74	204.87	134.12	10.03	4.36	3.35	0.00
42	Palaiokastro	Thesprotia	Mesokarpos	203.91	160.48	36.42	0.00	0.00	7.01	0.00
43	korwni	Thesprotia	Mesokarpos	387.11	237.21	118.11	11.81	9.69	6.76	3.53
44	Anthousa	Preveza	Lianolia	360.39	222.53	94.81	20.45	9.85	9.46	3.29
45	Parga	Preveza	Lianolia	502.39	272.52	169.34	24.95	26.06	6.26	3.27
46	Morfi	Thesprotia	Lianolia	525.28	319.74	161.40	17.96	16.20	6.55	3.42
47	Morfi	Thesprotia	Lianolia	248.42	202.48	31.53	9.96	4.46	0.00	0.00
48	Parga	Preveza	Lianolia	399.28	193.60	126.98	30.88	26.87	13.76	7.18
49	Anthousa	Preveza	Lianolia	375.08	215.17	107.55	21.18	23.46	7.72	0.00
50	Ag. Kuriaki	Preveza	Lianolia	174.31	102.09	29.95	21.86	12.45	7.97	0.00
51	Perdika	Thesprotia	Lianolia	682.96	343.71	243.18	44.97	37.57	13.53	0.00
52	Livadari	Thesprotia	Lianolia	492.85	258.18	160.67	26.93	34.61	12.46	0.00
53	Agia	Preveza	Lianolia	585.84	318.13	192.08	32.44	31.46	9.30	2.43
54	Agia	Preveza	Lianolia	621.82	302.73	215.52	34.90	49.81	14.00	4.87
55	Parga	Preveza	Lianolia	418.86	231.77	130.66	20.73	21.64	9.24	4.82
56	Anthousa	Preveza	Lianolia	295.45	164.60	90.52	13.18	18.35	8.81	0.00
57	Agia	Preveza	Lianolia	345.51	183.40	136.74	11.34	9.48	4.55	0.00
58	Ag. Kuriaki	Preveza	Lianolia	391.02	227.61	115.00	20.54	14.68	8.67	4.53
59	Morfi	Thesprotia	Lianolia	299.45	148.62	104.01	16.90	18.62	11.30	0.00
60	Parga	Preveza	Lianolia	238.79	126.83	78.48	11.86	14.30	7.32	0.00
61	Parga	Preveza	Lianolia	325.85	192.93	113.48	11.23	8.21	0.00	0.00
62	Morfi	Preveza	Lianolia	355.53	190.31	119.01	16.07	18.76	11.38	0.00
63	Agia	Preveza	Lianolia	281.64	159.45	93.52	14.45	11.32	2.90	0.00
64	Loutrotopos	Arta	Koroneiki	228.87	56.23	46.24	19.82	28.33	58.59	19.66
65	Kleidi	Arta	Table olives	224.42	59.76	52.97	6.54	21.45	52.43	31.28

66	Kompoti	Arta	Table olives	160.74	69.48	42.87	4.74	8.91	22.82	11.91
67	Kompoti	Arta	Table olives	245.99	135.81	64.76	5.35	9.30	21.44	9.33
68	Foteino	Arta	Table olives	143.19	43.41	36.55	7.93	14.91	25.45	14.94
69	Paliokatouno	Arta	Lianolia	254.03	74.67	70.53	15.16	19.78	51.67	22.22
70	Akropotamia	Arta	Koroneiki	146.11	61.02	34.89	7.16	12.14	21.53	9.37
71	Neoxwri	Arta	Lianolia	147.52	57.85	49.52	3.66	8.61	20.20	7.67
72	Kommeno	Arta	Table olives	215.85	90.47	65.20	6.77	10.11	29.11	14.19
73	Ano Petra	Arta	Table olives	112.08	40.06	23.58	5.64	11.79	24.25	6.75
74	Vlaxerna	Arta	Table olives	360.40	77.75	85.93	20.30	38.16	114.01	38.26
75	Foteino	Arta	Table olives	86.16	36.63	20.37	4.92	6.00	14.81	3.44
76	Makriniada	Arta	Table olives	68.23	37.84	5.69	7.70	6.70	10.30	0.00
77	Makriniada	Arta	Table olives	81.31	34.62	0.00	8.04	2.80	35.85	0.00
78	Agia	Preveza	Lianolia	397.17	216.91	132.02	14.89	15.55	12.30	5.50
79	Parga	Preveza	Lianolia	210.54	122.05	71.61	7.92	5.79	3.18	0.00
80	Agia	Preveza	Lianolia	369.52	193.20	124.76	19.70	20.58	11.29	0.00
81	Parga	Preveza	Lianolia	195.33	120.64	55.98	10.78	4.83	3.09	0.00
82	Parga	Preveza	Lianolia	201.28	116.20	67.62	8.62	4.85	3.99	0.00
83	Agia	Preveza	Lianolia	290.05	151.08	102.93	12.39	12.94	5.23	5.46
84	Anthousa	Preveza	Lianolia	179.24	115.79	46.37	7.38	7.00	2.69	0.00
85	Anthousa	Preveza	Lianolia	174.64	100.06	64.16	6.15	4.28	0.00	0.00
86	Parapotamos	Thesprotia	Lianolia	62.72	24.91	1.54	15.66	13.63	6.98	0.00
87	Vrisella filiatwn	Thesprotia	Agria	77.03	52.08	8.02	9.05	7.88	0.00	0.00
88	Palampas filiatwn	Thesprotia	Mesokarpos	231.60	125.59	15.71	44.29	40.09	5.92	0.00
89	Parapotamos	Thesprotia	Agria	197.96	119.14	31.05	28.29	14.07	5.40	0.00
90	Kourenta	Thesprotia	Koroneiki	405.21	150.69	115.88	40.79	63.90	22.31	11.65
91	Plaisko Filiatwn	Thesprotia	Lianolia	49.87	16.15	2.55	16.30	11.02	3.85	0.00
92	Elia Filiatwn	Thesprotia	Amfissis	152.63	54.44	26.63	23.67	29.47	14.61	3.81
93	Seleukia	Thesprotia	Kalamon	181.13	116.16	42.13	8.39	10.71	3.74	0.00
94	Petrovitsa	Thesprotia	Table olives	24.23	14.03	0.82	6.48	2.90	0.00	0.00
95	Kastri	Thesprotia	Lianolia	224.61	134.16	16.90	35.40	32.70	5.46	0.00
96	Dramesi	Thesprotia	Kalamon	138.94	50.40	19.49	20.76	38.27	4.90	5.12
97	Filiates	Thesprotia	Mesokarpos	291.83	175.90	73.57	16.60	20.22	5.55	0.00
98	Nea Seleukia	Thesprotia	Mesokarpos	247.46	193.43	37.31	10.98	5.73	0.00	0.00
99	Axladia filiatwn	Thesprotia	Mesokarpos	313.35	173.25	73.42	22.71	27.90	16.07	0.00
100	Marousi	Thesprotia	Kalamon	171.88	64.46	13.57	36.18	46.50	11.16	0.00
101	Crete	Crete	Koroneiki	207.71	15.09	25.60	24.89	53.03	59.91	29.19
102	Crete	Crete	Koroneiki	331.38	50.95	41.62	20.77	54.69	112.28	51.06
103	Ag. Kuriaki	Preveza	Lianolia	41.51	17.89	12.34	4.40	6.89	0.00	0.00
104	Agia	Preveza	Lianolia	130.30	81.51	26.58	12.95	9.25	0.00	0.00
105	Agia	Preveza	Lianolia	679.70	291.27	287.89	32.34	38.17	19.74	10.30
106	Parga	Preveza	Lianolia	67.50	34.81	17.73	8.00	6.96	0.00	0.00
107	Agia	Preveza	Lianolia	177.16	90.44	63.88	17.66	5.16	0.00	0.00
108	Anthousa	Preveza	Lianolia	29.21	11.65	7.10	4.37	6.08	0.00	0.00
109	Gardiki	Thesprotia	Kalamon	98.84	42.00	44.90	3.12	5.70	3.13	0.00
110	Agia	Preveza	Lianolia	117.59	64.55	50.68	0.72	1.64	0.00	0.00

111	Gardiki	Thesprotia	Kalamon	153.13	88.83	59.08	2.93	2.29	0.00	0.00
112	Anthousa	Preveza	Lianolia	47.67	25.74	12.25	4.37	5.32	0.00	0.00
113	Afra	Corfu	Lianolia	25.42	16.41	0.00	5.31	3.70	0.00	0.00
114	Kontokali	Corfu	Koroneiki	160.42	43.43	6.20	66.43	35.60	8.76	0.00
115	Gardelades	Corfu	Koroneiki	102.95	67.97	3.45	14.59	9.14	7.80	0.00
116	Palaiokastritsa	Corfu	Lianolia	18.93	12.95	0.00	5.98	0.00	0.00	0.00
117	Kalapoda	Corfu	Koroneiki	102.66	26.10	9.99	22.54	17.65	26.37	0.00
118	Liapades	Corfu	Lianolia	7.17	0.00	0.00	5.32	1.85	0.00	0.00
119	Gardelades	Corfu	Lianolia	198.22	137.74	44.09	7.65	3.00	5.75	0.00
120	Louros	Preveza	Lianolia	487.35	227.93	159.59	16.69	19.92	38.26	24.97
121	Kamamarina	Preveza	Lianolia	269.28	124.78	49.12	13.25	8.81	53.15	20.18
122	Nea Sinwpi	Preveza	Lianolia	419.93	137.31	126.85	15.25	20.82	70.57	49.12
123	Wrwpos	Preveza	Koroneiki	69.24	18.79	15.62	5.87	9.81	11.78	7.38
124	Papadates	Preveza	Lianolia	118.99	77.02	20.27	7.22	0.00	14.48	0.00
125	Nea kerasounta	Preveza	Koroneiki	270.31	78.08	50.12	18.09	22.43	58.96	42.62
126	Kotsanopoulo	Preveza	Koroneiki	142.25	44.18	11.63	9.84	10.27	52.61	13.73
127	Galatas	Preveza	Amfissis	113.05	77.96	6.68	6.46	4.50	12.95	4.51
128	Stefani	Preveza	Koroneiki	161.77	98.77	38.58	9.67	5.05	9.70	0.00
129	Wrwpos	Preveza	Lianolia	77.10	54.07	1.96	7.75	0.00	13.32	0.00
130	Nikolitsi	Preveza	Amfissis	81.36	29.93	6.30	7.11	0.00	38.02	0.00
131	Wrwpos	Preveza	Lianolia	250.58	127.60	59.92	11.65	18.26	23.37	9.76
132	Vlaxerna	Arta	Amfissis	50.59	27.66	8.03	2.27	3.55	9.09	0.00
133	Mirsini	Preveza	Koroneiki	229.23	89.54	43.03	10.40	10.86	46.36	29.04
134	Wrwpos	Preveza	Kalamon	11.54	4.72	0.00	4.48	2.34	0.00	0.00
135	Faskomhlia	Thesprotia	Mesokarpos	275.65	37.66	12.07	35.97	61.93	97.49	30.54
136	Mesovouni	Thesprotia	Table olives	153.79	111.80	36.40	2.74	2.86	0.00	0.00
137	Parga	Preveza	Lianolia	153.39	96.72	44.36	12.31	0.00	0.00	0.00
138	Spatharaioi	Thesprotia	Table olives	335.90	147.75	85.86	21.11	28.53	37.35	15.60
139	Palioxwri	Thesprotia	Table olives	146.83	95.06	38.66	6.41	6.70	0.00	0.00
140	Suvota (vouno)	Thesprotia	Lianolia	286.78	226.55	40.66	12.35	3.68	3.54	0.00
141	Tzara	Thesprotia	Lianolia	481.77	298.97	89.91	25.36	41.93	21.19	4.42
142	Agia	Preveza	Koroneiki	221.32	99.15	81.53	8.28	21.13	7.38	3.85
143	Faskomhlia	Thesprotia	Mesokarpos	182.15	69.48	45.36	8.35	25.08	25.13	8.75
144	Margariti	Thesprotia	Lianolia	271.42	153.24	102.31	9.36	6.51	0.00	0.00
145	Kodra Mazarakias	Thesprotia	Mesokarpos	63.57	17.52	15.98	19.42	2.17	5.56	2.90
146	Faskomhlia	Thesprotia	Mesokarpos	42.09	11.61	9.16	3.45	15.29	2.59	0.00
147	Skorpiwna	Thesprotia	Mesokarpos	261.32	28.65	37.70	31.19	68.83	68.23	26.72
148	Megarxi	Arta	Table olives	75.72	47.09	5.51	7.25	7.57	8.31	0.00
149	Kompoti	Arta	Table olives	246.30	98.86	76.91	8.93	22.65	25.59	13.36
150	Peta	Arta	Table olives	68.66	43.42	10.63	4.80	5.01	4.81	0.00
151	Korvovouni	Arta	Table olives	195.59	75.43	41.97	11.52	20.04	35.92	10.72
152	Sellades	Arta	Table olives	229.00	62.32	52.69	13.34	31.67	48.66	20.32
153	Gramenitsa	Arta	Table olives	25.92	19.12	0.00	4.01	2.79	0.00	0.00
154	Dimario	Arta	Table olives	134.21	37.25	40.29	6.14	20.53	19.71	10.29
155	Kleisto	Arta	Table olives	21.30	9.79	2.29	2.58	4.05	2.59	0.00

156	Xrusaugi Souliou	Thesprotia	Table olives	124.97	82.48	17.16	10.25	5.95	9.14	0.00
157	Tzara	Thesprotia	Lianolia	231.99	71.50	44.33	34.89	38.87	32.66	9.74
158	Zervoxwri Souliou	Thesprotia	Koroneiki	21.48	14.20	0.00	6.02	1.26	0.00	0.00
159	Mandrotopos Skandalou	Thesprotia	Koroneiki	390.35	92.89	80.00	33.84	58.90	87.98	36.75
160	Gardiki	Thesprotia	Kalamon	1100.12	862.18	101.41	61.87	23.16	51.50	0.00
161	Perixati	Thesprotia	Table olives	443.02	109.48	40.33	63.70	77.38	130.35	21.78
162	Pagkrati	Thesprotia	Lianolia	154.50	76.82	36.65	10.95	15.24	9.76	5.09
163	Karvounari	Thesprotia	Agria	177.01	93.05	47.24	19.50	6.79	10.43	0.00
164	Mouzakeika	Thesprotia	Lianolia	447.62	187.21	148.37	26.15	43.70	36.71	5.48
165	Prodromi Souliou	Thesprotia	Table olives	400.72	144.18	104.33	24.07	74.02	42.92	11.20
166	Kanalaki	Preveza	Lianolia	322.73	204.53	98.57	15.81	3.83	0.00	0.00
167	Gliki	Thesprotia	Kalamon	318.56	267.60	33.95	6.21	10.81	0.00	0.00
168	Aidoni Fanariou	Thesprotia	Lianolia	469.30	260.78	140.74	23.65	25.87	13.55	4.72
169	Prodromi Souliou	Thesprotia	Lianolia	309.24	178.47	79.39	20.18	21.08	10.12	0.00
170	Souli	Thesprotia	Agria	197.47	108.46	38.05	16.69	19.92	14.35	0.00
171	Agios Lewn	Zakynthos	Lianolia	23.43	1.86	0.00	8.85	12.71	0.00	0.00
172	Agios Lewn	Zakynthos	Koroneiki	519.89	178.98	157.84	45.95	57.58	50.68	28.87
173	Maries	Zakynthos	Lianolia	27.46	4.05	0.00	9.61	13.80	0.00	0.00
174	Maries	Zakynthos	Koroneiki	513.78	160.88	152.82	80.37	34.06	49.06	36.59
175	Kampi	Zakynthos	Koroneiki	485.52	169.08	129.34	50.19	51.19	51.50	34.22
176	Kampi	Zakynthos	Lianolia	27.48	4.24	0.00	10.08	13.16	0.00	0.00
177	Morena	Messinia	Koroneiki	86.27	3.20	9.27	32.31	33.74	4.76	2.99
178	Agrilos	Messinia	Koroneiki	296.92	94.83	76.73	47.90	7.02	38.76	31.68
179	Giolaka	Messinia	Koroneiki	203.45	106.93	42.21	21.16	33.15	0.00	0.00
180	Zaliari	Messinia	Koroneiki	98.20	57.94	6.28	20.24	13.74	0.00	0.00
181	Galatova	Messinia	Koroneiki	138.53	38.15	25.10	19.25	33.11	18.17	4.74
182	Plakwti	Thesprotia	Table olives	233.71	100.72	8.38	6.57	19.21	15.81	11.01
183	Rizani	Thesprotia	Koroneiki	423.29	97.01	143.83	26.01	85.36	34.77	36.31
184	Ragio	Thesprotia	Koroneiki	291.28	137.04	106.74	15.46	18.69	6.53	6.82
185	Trikorifo	Thesprotia	Kalamon	1039.24	712.06	207.67	33.30	28.56	47.70	9.96
186	Aetos	Thesprotia	Koroneiki	67.84	19.56	10.79	9.96	23.10	4.44	0.00
187	Graikoxwri	Thesprotia	Mesokarpos	476.75	54.20	134.30	21.45	134.41	53.78	78.61
188	Filippiada	Arta	Koroneiki	155.84	15.75	8.57	16.76	33.67	59.48	21.60
189	Vigla	Arta	Koroneiki	227.14	86.11	57.14	17.78	30.18	29.72	6.21
190	Peta	Arta	Amfissis	2.68	0.00	0.00	1.76	0.92	0.00	0.00
191	Korvovouni	Arta	Amfissis	32.00	11.03	0.00	6.55	14.43	0.00	0.00
192	Amfiloxia	Arta	Kalamon	1.32	0.00	0.00	0.65	0.68	0.00	0.00
193	Kleidi	Arta	Table olives	154.11	91.10	51.07	7.64	4.30	0.00	0.00
194	Vlaxerna	Arta	Amfissis	123.60	42.37	29.11	6.99	16.78	21.02	7.32
195	Zigos	Arta	Amfissis	180.63	23.45	21.26	20.11	31.50	71.36	12.96
196	Paχikalamos	Arta	Koroneiki	256.13	85.76	93.88	16.29	35.45	19.06	5.69
197	Preveza	Preveza	Lianolia	139.42	55.75	63.24	4.41	9.98	2.95	3.08
198	Kentriko	Arta	Koroneiki	245.15	85.36	79.32	19.80	43.00	12.70	4.97

Table S5. Test of Kruskal Wallis H, Average (Ave) and Median (Med) of ligstroside aglycone (**5**) and oleuropein aglycone (**6**) in relation to the olive variety. Values of Ave and Med are expressed in mg/Kg.

	ligstroside aglycone (5)				oleuropein aglycone (6)			
**Variety**	**N**	**Ave**	**Med.**		**N**	**Ave**	**Med.**	
Amfissis	34	12 ± 2	8	Kruskal	34	11 ± 2	6	Kruskal
Koroneiki	26	16 ± 2	15	Wallis	26	26 ± 4	22	Wallis
Lianolia	63	16 ± 1	15	*18.63*	63	16 ± 2	14	*13.79*
Mesokarpos	14	17 ± 3	14	df	14	30 ± 10	18	df
Table olives	27	11 ± 2	7	*6*	27	17 ± 4	9	*6*
Kalamon	10	18 ± 6	7	sig *0.005*	10	17 ± 5	11	sig *0.032*
Agria	4	18 ± 4	18		4	12 ± 3	11	

Ave = Average; Med. = Median; df = degree of freedom; sig = significance

Table S6. Test of Kruskal Wallis H, Average (Ave) and Median (Med) of oleokoronal (**7**) and oleomissional (**8**) in relation to the olive variety. Values of Ave and Med are expressed in mg/Kg.

	oleokoronal (7)				oleomissional (8)			
**Variety**	**N**	**Ave**	**Med.**		**N**	**Ave**	**Med.**	
Amfissis	34	27 ± 5	15	Kruskal	34	6 ± 2	1	Kruskal
Koroneiki	26	33 ± 6	24	Wallis	26	14 ± 3	8	Wallis
Lianolia	63	13 ± 2	8	*21.13*	63	4 ± 1	-	*27.39*
Mesokarpos	14	22 ± 8	6	df	14	11 ± 6	-	df
Table olives	27	27 ± 6	21	*6*	27	9 ± 2	9	*6*
Kalamon	10	12 ± 6	3	sig *0.002*	10	2 ± 1	-	sig *< 0.001*
Agria	4	8 ± 3	8		4	-	-	

Ave = Average; Med. = Median; df = degree of freedom; sig = significance

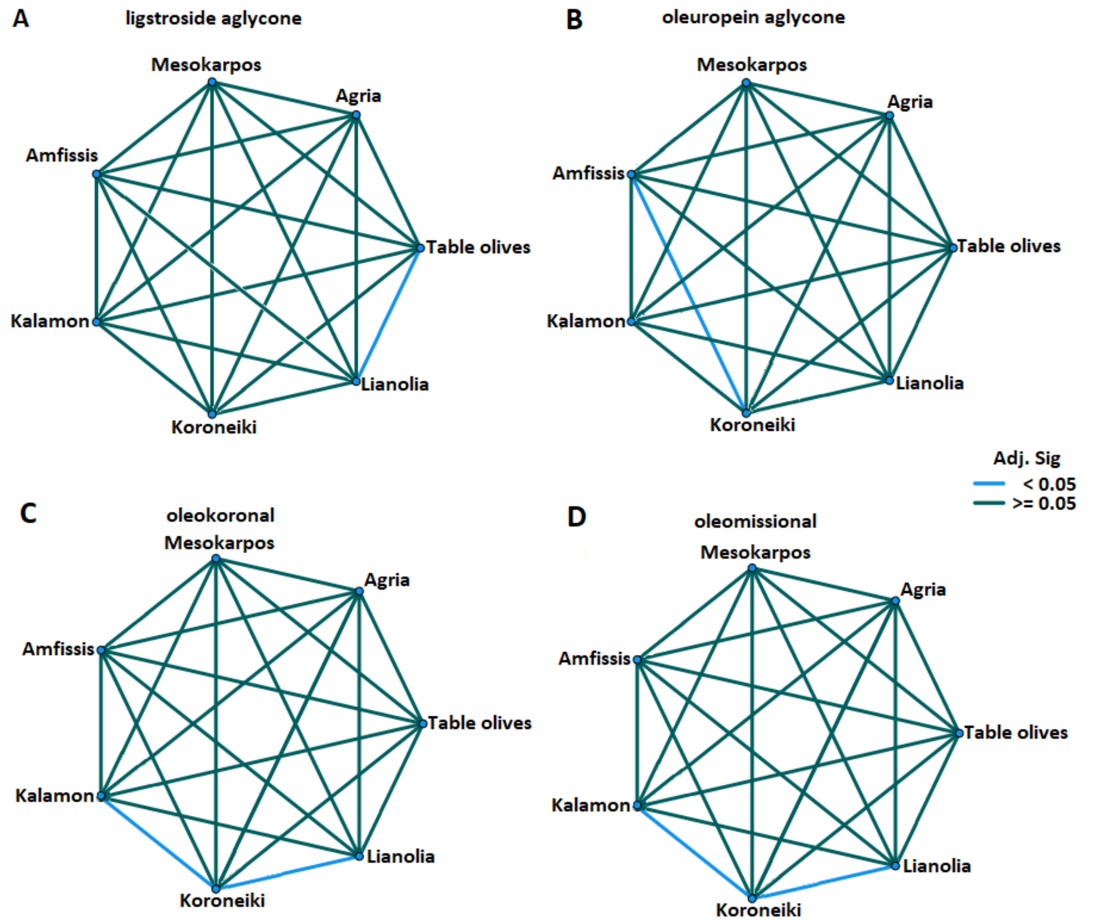

Figure S1. Pairwise Comparisons of Variety. A) Average of ligstroside aglycone (**5**); B) Average of oleuropein aglycone (**6**); C) Average of oleokoronal (**7**); D) Average of oleomissional (**8**). Significance values have been adjusted by the Bonferroni correction for multiple tests.

Table S7. Test of Kruskal Wallis H, Average (Ave) and Median (Med) of ligstroside aglycone (**5**), among the month of olive oil production. Values of Ave and Med are expressed in mg/Kg.

		ligstroside aglycone (5)		
Harvest Month	N	Ave	Med.	
October	20	18 ± 3	15	Kruskal Wallis
November	99	16 ± 1	14	*9.44*
December	41	14 ± 2	10	df = *3*
January to March	18	9 ± 2	7	sig = *0.024*

Ave = Average; Med. = Median; df = degree of freedom; sig = significance

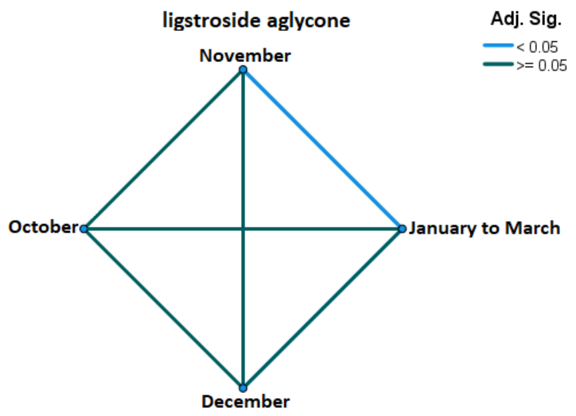

Figure S2. Pairwise Comparisons of harvest month of average of ligstroside aglycone (**5**). Significance values have been adjusted by the Bonferroni correction for multiple tests.

Table S8. Test of Kruskal Wallis H, Average (Ave) and Median (Med) of ligstroside aglycone (**5**), oleuropein aglycone (**6**) and oleokoronal (**7**) among prefectures of Epirus. Values of Ave and Med are expressed in mg/Kg.

	ligstroside aglycone (5)				oleuropein aglycone (6)				oleokoronal (7)			
**Prefecture**	**N**	**Ave**	**Med.**		**N**	**Ave**	**Med.**		**N**	**Ave**	**Med.**	
Arta	62	11 ± 1	8	Kruskal	62	14 ± 2	10	Kruskal	62	28 ± 4	21	Kruskal
Preveza	52	14 ± 1	12	Wallis	52	14 ± 2	10	Wallis	52	16 ± 3	9	Wallis
				17.29				*7.90*				*9.60*
Thesprotia	63	19 ± 2	16	df = 2	63	25 ± 3	19	df = 2	63	19 ± 3	8	df = 2
				sig < *0.001*				sig = *0.019*				sig = *0.008*

Ave = Average; Med. = Median; df = degree of freedom; sig = significance

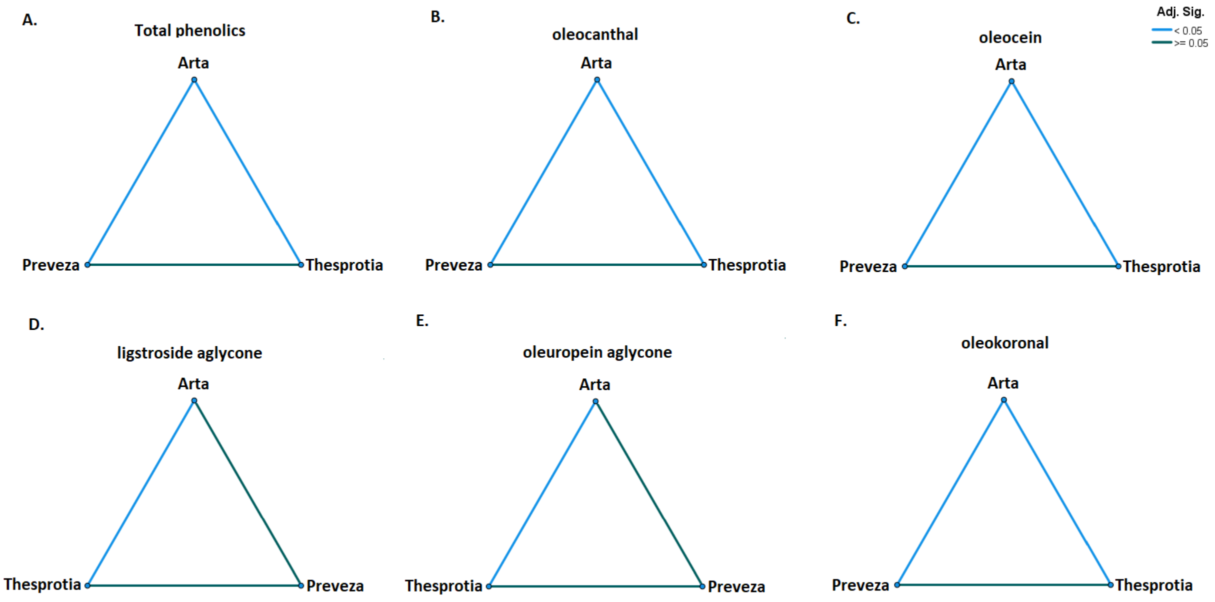

Figure S3. Pairwise Comparisons of prefecture. A) Average of total phenolics; B) Average of oleocanthal (**3**); C) Average of oleocein (**4**); D) Average of ligstroside aglycone (**5**); E) Average of oleuropein aglycone (**6**); F) Average of oleokoronal (**7**). Significance values have been adjusted by the Bonferroni correction for multiple tests.

Table S9: Origins, altitude, month of harvest and analysis and variety of olive oil samples.

Sample	Geographical factor		Climatological factors						Sample details		Variety
	Town	Province	Altitude (m)	Altitude class*	Average Temperature (°C)	Temperature class¥	Average Rainfall (mm)	Rainfall class¤	Harvest month	Analysis month	
1	Foteino	Arta	511	2	14.5	1	66.6	1	October	March	Amfissis
2	Petra	Arta	548	2	14.2	1	66.6	1	October	March	Amfissis
3	Glikorizo	Arta	36	1	17.7	2	66.6	1	October	March	Amfissis
4	Neoxwri	Arta	10	1	17.8	2	66.6	1	October	March	Amfissis
5	Menidi	Arta	12	1	17.7	2	66.6	1	October	March	Koroneiki
6	Megarxi	Arta	107	1	17.1	2	66.0	1	October	March	Amfissis
7	Foteino	Arta	511	2	14.5	1	66.6	1	October	March	Amfissis
8	Dimario	Arta	604	2	13.5	1	66.6	1	October	March	Amfissis
9	Kompoti	Arta	71	1	17.2	2	66.6	1	October	March	Amfissis
10	Kleisto	Arta	433	2	15.2	1	68.3	1	October	March	Amfissis
11	Neoxwraki	Arta	86	1	17.2	2	66.6	1	October	March	Amfissis
12	Foteino	Arta	511	2	14.5	1	66.6	1	October	March	Amfissis
13	Menidi	Arta	12	1	17.7	2	66.6	1	October	March	Amfissis
14	Petra	Arta	548	2	14.2	1	66.6	1	October	March	Amfissis
15	Anoixiatiko	Arta	12	1	17.8	2	66.6	1	October	March	Amfissis
16	Menidi	Arta	12	1	17.7	2	66.6	1	November	March	Amfissis
17	Foteino	Arta	511	2	14.5	1	66.6	1	November	March	Amfissis
18	Kleidi	Arta	563	2	14.1	1	66.6	1	November	March	Amfissis
19	Gorgomilos	Preveza	689	2	13.7	1	68.3	1	November	March	Koroneiki
20	Loutropoulo	Arta	4	1	17.8	2	66.6	1	November	March	Koroneiki
21	Petra	Arta	548	2	14.2	1	66.6	1	November	March	Amfissis
22	Makriniada	Arta	393	2	15.7	1	68.3	1	November	March	Amfissis
23	Paliokatouno	Arta	348	2	15.8	1	68.3	1	November	March	Amfissis
24	Petra	Arta	548	2	14.2	1	66.6	1	November	March	Amfissis
25	Neoxwraki	Arta	86	1	17.2	2	66.6	1	November	March	Amfissis
26	Loutrotopos	Arta	4	1	17.8	2	66.6	1	November	March	Amfissis
27	Paxikalamos	Arta	5	1	17.8	2	66.6	1	November	March	Koroneiki
28	Kompoti	Arta	71	1	17.2	2	66.6	1	November	March	Amfissis
29	Katw Athnamanio	Arta	450	2	16.4	1	68.3	1	November	March	Koroneiki
30	Megarxi	Arta	107	1	17.1	2	66.0	1	November	March	Amfissis
31	Parga	Preveza	11	1	17.5	2	71.6	2	November	March	Koroneiki
32	Tzara	Thesprotia	34	1	17.3	2	71.6	2	November	March	Lianolia
33	Faskomilia	Thesprotia	41	1	16.5	2	66.0	1	November	March	Koroneiki
34	Eleutheri	Thesprotia	181	2	16.5	2	71.6	2	November	March	Lianolia
35	Margariti	Thesprotia	170	2	16.5	2	71.6	2	November	March	Mesokarpos
36	Katavothra	Thesprotia	114	1	16.8	2	71.6	2	November	March	Lianolia
37	Themelo	Thesprotia	24	1	17.4	2	71.6	2	November	March	Lianolia
38	Ag. Kuriaki	Preveza	208	2	16.1	1	71.6	2	November	March	Koroneiki
39	Morfi	Thesprotia	155	2	16.5	2	71.6	2	November	March	Table olives
40	Eleutheri	Thesprotia	181	2	16.5	2	71.6	2	November	March	Lianolia

41	Palaiokastro	Thesprotia	142	1	16.6	2	71.6	2	November	March	Mesokarpos
42	Palaiokastro	Thesprotia	142	1	16.6	2	71.6	2	November	March	Mesokarpos
43	korwni	Thesprotia	39	1	17.2	2	71.6	2	November	March	Mesokarpos
44	Anthousa	Preveza	190	2	16.3	1	71.6	2	November	March	Lianolia
45	Parga	Preveza	11	1	17.5	2	71.6	2	November	March	Lianolia
46	Morfi	Thesprotia	155	2	16.5	2	71.6	2	November	March	Lianolia
47	Morfi	Thesprotia	155	2	16.5	2	71.6	2	November	March	Lianolia
48	Parga	Preveza	11	1	17.5	2	71.6	2	November	March	Lianolia
49	Anthousa	Preveza	190	2	16.3	1	71.6	2	November	April	Lianolia
50	Ag. Kuriaki	Preveza	208	2	16.1	1	71.6	2	November	April	Lianolia
51	Perdika	Thesprotia	245	2	15.9	1	71.6	2	November	April	Lianolia
52	Livadari	Thesprotia	296	2	15.6	1	71.6	2	November	April	Lianolia
53	Agia	Preveza	427	2	14.8	1	71.6	2	November	April	Lianolia
54	Agia	Preveza	427	2	14.8	1	71.6	2	November	April	Lianolia
55	Parga	Preveza	11	1	17.5	2	71.6	2	November	April	Lianolia
56	Anthousa	Preveza	190	2	16.3	1	71.6	2	November	April	Lianolia
57	Agia	Preveza	427	2	14.8	1	71.6	2	November	April	Lianolia
58	Ag. Kuriaki	Preveza	208	2	16.1	1	71.6	2	November	April	Lianolia
59	Morfi	Thesprotia	155	2	16.5	2	71.6	2	November	April	Lianolia
60	Parga	Preveza	11	1	17.5	2	71.6	2	November	April	Lianolia
61	Parga	Preveza	11	1	17.5	2	71.6	2	November	April	Lianolia
62	Morfi	Preveza	155	2	16.5	2	71.6	2	November	April	Lianolia
63	Agia	Preveza	427	2	14.8	1	71.6	2	November	April	Lianolia
64	Loutrotopos	Arta	4	1	17.8	2	66.6	1	November	April	Koroneiki
65	Kleidi	Arta	563	2	14.1	1	66.6	1	November	April	Table olives
66	Kompoti	Arta	71	1	17.2	2	66.6	1	November	April	Table olives
67	Kompoti	Arta	71	1	17.2	2	66.6	1	November	April	Table olives
68	Foteino	Arta	511	2	14.5	1	66.6	1	December	April	Table olives
69	Paliokatouno	Arta	348	2	15.8	1	68.3	1	November	April	Lianolia
70	Akropotamia	Arta	6	1	15.8	1	68.3	1	November	April	Koroneiki
71	Neoxwri	Arta	10	1	17.8	2	66.6	1	November	April	Lianolia
72	Kommeno	Arta	13	1	17.8	2	66.6	1	December	April	Table olives
73	Ano Petra	Arta	550	2	14.2	1	66.6	1	November	April	Table olives
74	Vlaxerna	Arta	37	1	17.8	2	68.3	1	November	April	Table olives
75	Foteino	Arta	511	2	14.5	1	66.6	1	November	April	Table olives
76	Makriniada	Arta	393	2	15.7	1	68.3	1	November	April	Table olives
77	Makriniada	Arta	393	2	15.7	1	68.3	1	November	April	Table olives
78	Agia	Preveza	427	2	14.8	1	71.6	2	November	April	Lianolia
79	Parga	Preveza	11	1	17.5	2	71.6	2	November	April	Lianolia
80	Agia	Preveza	427	2	14.8	1	71.6	2	November	April	Lianolia
81	Parga	Preveza	11	1	17.5	2	71.6	2	November	April	Lianolia
82	Parga	Preveza	11	1	17.5	2	71.6	2	November	April	Lianolia
83	Agia	Preveza	427	2	14.8	1	71.6	2	November	April	Lianolia
84	Anthousa	Preveza	190	2	16.3	1	71.6	2	November	April	Lianolia
85	Anthousa	Preveza	190	2	16.3	1	71.6	2	November	April	Lianolia

86	Parapotamos	Thesprotia	115	1	15.8	1	66.0	1	December	April	Lianolia
87	Vrisella filiatwn	Thesprotia	120	1	15.6	1	66.0	1	December	April	Agria
88	Palampas filiatwn	Thesprotia	335	2	14.7	1	66.0	1	December	May	Mesokarpos
89	Parapotamos	Thesprotia	115	1	15.8	1	66.0	1	December	May	Agria
90	Kourenta	Thesprotia	391	2	15.7	1	81.8	2	December	April	Koroneiki
91	Plaisko Filiatwn	Thesprotia	410	2	14.0	1	66.0	1	December	April	Lianolia
92	Elia Filiatwn	Thesprotia	128	1	15.9	1	66.0	1	December	April	Amfissis
93	Seleukia	Thesprotia	30	1	16.0	1	66.0	1	December	April	Kalamon
94	Petrovitsa	Thesprotia	523	2	14.7	1	81.8	2	December	April	Table olives
95	Kastri	Thesprotia	185	2	15.7	1	66.0	1	December	May	Lianolia
96	Dramesi	Thesprotia	194	2	14.0	1	66.0	1	December	May	Kalamon
97	Filiates	Thesprotia	200	2	15.3	1	66.0	1	December	May	Mesokarpos
98	Nea Seleukia	Thesprotia	30	1	16.0	1	66.0	1	December	April	Mesokarpos
99	Axladia filiatwn	Thesprotia	440	2	14.0	1	71.6	2	December	May	Mesokarpos
100	Marousi	Thesprotia	160	2	16.0	1	66.0	1	December	May	Kalamon
101	Crete	Crete	na	-	n.c.	-	n.c.	-	December	April	Koroneiki
102	Crete	Crete	na	-	n.c.	-	n.c.	-	December	April	Koroneiki
103	Ag. Kuriaki	Preveza	208	2	16.1	1	71.6	2	December	April	Lianolia
104	Agia	Preveza	427	2	14.8	1	71.6	2	December	May	Lianolia
105	Agia	Preveza	427	2	14.8	1	71.6	2	December	May	Lianolia
106	Parga	Preveza	11	1	17.5	2	71.6	2	December	May	Lianolia
107	Agia	Preveza	427	2	14.8	1	71.6	2	December	May	Lianolia
108	Anthousa	Preveza	190	2	16.3	1	71.6	2	December	April	Lianolia
109	Gardiki	Thesprotia	200	2	16.9	2	71.6	2	December	April	Kalamon
110	Agia	Preveza	427	2	14.8	1	71.6	2	December	April	Lianolia
111	Gardiki	Thesprotia	200	2	16.9	2	71.6	2	December	April	Kalamon
112	Anthousa	Preveza	190	2	16.3	1	71.6	2	December	April	Lianolia
113	Afra	Corfu	61	1	n.c.	-	n.c.	-	November	April	Lianolia
114	Kontokali	Corfu	4	1	n.c.	-	n.c.	-	November	April	Koroneiki
115	Gardelades	Corfu	149	2	n.c.	-	n.c.	-	November	April	Koroneiki
116	Palaiokastritsa	Corfu	29	1	n.c.	-	n.c.	-	November	April	Lianolia
117	Kalapoda	Corfu	na	-	n.c.	-	n.c.	-	November	April	Koroneiki
118	Liapades	Corfu	118	1	n.c.	-	n.c.	-	November	April	Lianolia
119	Gardelades	Corfu	149	1	n.c.	-	n.c.	-	November	April	Lianolia
120	Louros	Preveza	16	1	17.5	2	71.6	2	November	April	Lianolia
121	Kamamarina	Preveza	336	2	15.4	1	71.6	2	December	April	Lianolia
122	Nea Sinwpi	Preveza	43	1	17.2	2	71.6	2	November	April	Lianolia
123	Wrwpos	Preveza	33	1	23.0	2	71.6	2	December	April	Koroneiki
124	Papadates	Preveza	291	2	15.7	1	71.6	2	December	April	Lianolia
125	Nea kerasounta	Preveza	25	1	17.1	2	66.6	1	December	April	Koroneiki
126	Kotsanopoulo	Preveza	185	2	16.8	2	71.6	2	December	April	Koroneiki
127	Galatas	Preveza	189	2	16.3	1	71.6	2	December	April	Amfissis
128	Stefani	Preveza	15	1	17.3	2	71.6	2	November	April	Koroneiki
129	Wrwpos	Preveza	33	1	16.7	2	71.6	2	December	April	Lianolia
130	Nikolitsi	Preveza	243	2	15.9	1	71.6	2	December	April	Amfissis

131	Wrwpos	Preveza	33	1	16.7	2	71.6	2	December	April	Lianolia
132	Vlaxerna	Arta	40	1	17.8	2	68.3	1	December	April	Amfissis
133	Mirsini	Preveza	212	2	16.1	1	71.6	2	December	April	Koroneiki
134	Wrwpos	Preveza	33	1	16.7	2	71.6	2	December	April	Kalamon
135	Faskomhlia	Thesprotia	41	1	16.5	2	66.0	1	November	April	Mesokarpos
136	Mesovouni	Thesprotia	314	2	14.8	1	71.6	2	November	May	Table olives
137	Parga	Preveza	11	1	17.5	2	71.6	2	November	April	Lianolia
138	Spatharaioi	Thesprotia	135	1	16.7	2	71.6	2	November	May	Table olives
139	Palioxwri	Thesprotia	240	2	16.7	2	81.8	2	November	May	Table olives
140	Suvota (vouno)	Thesprotia	420	2	16.7	2	66	1	November	April	Lianolia
141	Tzara	Thesprotia	34	1	17.3	2	71.6	2	November	May	Lianolia
142	Agia	Preveza	427	2	14.8	1	71.6	2	November	April	Koroneiki
143	Faskomhlia	Thesprotia	41	1	16.5	2	66.0	1	November	May	Mesokarpos
144	Margariti	Thesprotia	170	2	16.5	2	71.6	2	November	May	Lianolia
145	Kodra Mazarakias	Thesprotia	211	2	16.0	1	71.6	2	January	April	Mesokarpos
146	Faskomhlia	Thesprotia	41	1	16.5	2	66.0	1	December	April	Mesokarpos
147	Skorpiwna	Thesprotia	71	1	16.2	1	71.6	2	December	May	Mesokarpos
148	Megarxi	Arta	107	1	17.1	2	66.0	1	January	April	Table olives
149	Kompoti	Arta	71	1	17.2	2	66.6	1	January	April	Table olives
150	Peta	Arta	174	2	17.0	2	68.3	1	January	April	Table olives
151	Korvovouni	Arta	571	2	14.5	1	68.3	1	January	April	Table olives
152	Sellades	Arta	80	1	17.3	2	66.6	1	January	April	Table olives
153	Gramenitsa	Arta	58	1	17.8	2	68.3	1	January	April	Table olives
154	Dimario	Arta	604	2	13.5	1	66.6	1	January	April	Table olives
155	Kleisto	Arta	433	2	15.2	1	68.3	1	January	April	Table olives
156	Xrusaugi Souliou	Thesprotia	206	2	16.3	1	71.6	2	October	April	Table olives
157	Tzara	Thesprotia	34	1	17.3	2	71.6	2	November	April	Lianolia
158	Zervoxwri Souliou	Thesprotia	314	2	16.2	1	71.6	2	January	April	Koroneiki
159	Mandrotopos Skandalou	Thesprotia	51	1	17.2	2	71.6	2	October	April	Koroneiki
160	Gardiki	Thesprotia	200	2	16.9	2	71.6	2	October	April	Kalamon
161	Perixati	Thesprotia	40	1	17.2	2	71.6	2	November	April	Table olives
162	Pagkrati	Thesprotia	112	1	16.7	2	71.6	2	November	April	Lianolia
163	Karvounari	Thesprotia	140	1	16.7	2	71.6	2	November	April	Agria
164	Mouzakeika	Thesprotia	67	1	17.0	2	71.6	2	November	April	Lianolia
165	Prodromi Souliou	Thesprotia	210	2	16.1	1	71.6	2	November	April	Table olives
166	Kanalaki	Preveza	19	1	17.4	2	71.6	2	November	April	Lianolia
167	Gliki	Thesprotia	56	1	17.2	2	71.6	2	November	April	Kalamon
168	Aidoni Fanariou	Thesprotia	70	1	17.0	2	71.6	2	November	April	Lianolia
169	Prodromi Souliou	Thesprotia	210	2	16.1	1	71.6	2	November	April	Lianolia
170	Souli	Thesprotia	579	2	13.7	1	71.6	2	November	April	Agria
171	Agios Lewn	Zakynthos	373	2	n.c.	-	n.c.	-	November	April	Lianolia
172	Agios Lewn	Zakynthos	373	2	n.c.	-	n.c.	-	November	April	Koroneiki
173	Maries	Zakynthos	384	2	n.c.	-	n.c.	-	December	April	Lianolia
174	Maries	Zakynthos	384	2	n.c.	-	n.c.	-	December	April	Koroneiki

175	Kampi	Zakynthos	169	2	n.c.	-	n.c.	-	December	April	Koroneiki
176	Kampi	Zakynthos	169	2	n.c.	-	n.c.	-	December	April	Lianolia
177	Morena	Messinia	65	1	n.c.	-	n.c.	-	November	April	Koroneiki
178	Agrilos	Messinia	67	1	n.c.	-	n.c.	-	November	April	Koroneiki
179	Giolaka	Messinia	66	1	n.c.	-	n.c.	-	November	April	Koroneiki
180	Zaliari	Messinia	60	1	n.c.	-	n.c.	-	December	April	Koroneiki
181	Galatova	Messinia	65	1	n.c.	-	n.c.	-	December	April	Koroneiki
182	Plakwti	Thesprotia	380	2	15.8	1	81.8	2	November	April	Table olives
183	Rizani	Thesprotia	150	1	14.6	1	81.8	2	January	April	Koroneiki
184	Ragio	Thesprotia	150	1	16.5	2	66.0	1	November	May	Koroneiki
185	Trikorifo	Thesprotia	265	1	14.8	1	66.0	1	November	April	Kalamon
186	Aetos	Thesprotia	160	2	17.6	2	81.8	2	January	April	Koroneiki
187	Graikoxwri	Thesprotia	455	2	16.4	1	66.0	1	November	April	Mesokarpos
188	Filippiada	Arta	20	1	18.0	2	68.3	1	January	April	Koroneiki
189	Vigla	Arta	3	1	17.8	2	66.6	1	November	April	Koroneiki
190	Peta	Arta	174	2	17.0	2	68.3	1	March	April	Amfissis
191	Korvovouni	Arta	571	2	14.5	1	68.3	1	March	May	Amfissis
192	Amfiloxia	Arta	80	1	17.2	2	66.6	1	March	April	Kalamon
193	Kleidi	Arta	563	2	14.1	1	66.6	1	October	April	Table olives
194	Vlaxerna	Arta	40	1	17.8	2	68.3	1	November	April	Amfissis
195	Zigos	Arta	491	2	15.1	1	68.3	1	January	April	Amfissis
196	Paχikalamos	Arta	5	1	17.8	2	66.6	1	October	April	Koroneiki
197	Preveza	Preveza	8	1	17.8	2	66.6	1	March	April	Lianolia
198	Kentriko	Arta	440	2	15.1	1	68.3	1	November	April	Koroneiki

na = not available nc = no calculate; *1 = ≤ 150 m, ¥1 = < 16.5 ºC, ¤1 = < 70 mm; *2 = > 150 m, ¥2 = ≥ 16.5 ºC, ¤2 = > 70 mm

Table S10. Results of Mann-Whitney U test between the two classes of altitude.

	Total phenolics	Oleocanthal (3)	Oleocein (4)	ligstroside aglycone (5)	oleuropein aglycone (6)	oleokoronal (7)	oleomissional (8)
Mann-Whitney U	3865	3735	3588	3901	3922	3865	3390
Asymp. Sig. (2-tailed)	0.853	0.571	0.319	0.936	0.985	0.853	0.093

Table S11. Results of Mann-Whitney U test and Average (Ave) of total phenolics, oleocanthal (**3**), oleocein (**4**), ligstroside aglycone (**5**), oleuropein aglycone (**6**), oleokoronal (**7**) and oleomissional (**8**) among the two classes of average rainfall. Values of Ave are expressed in mg/Kg.

	Total phenolics	Oleocanthal (3)	Oleocein (4)	ligstroside aglycone (5)	oleuropein aglycone (6)	oleokoronal (7)	oleomissional (8)
Ave of class 1 (< 70 mm)	202 ± 16	91 ± 10	45 ± 5	14 ± 1	18 ± 2	26 ± 3	9 ± 1
Ave of class 2 (> 70 mm)	287 ± 19	151 ± 12	80 ± 6	16 ± 1	18 ± 2	17 ± 2	5 ± 1
Mann-Whitney U	5176	5688	5483	4546	3890	3085	3265
Asymp. Sig. (2-tailed)	< 0.001	< 0.001	< 0.001	0.086	0.848	0.011	0.032

Table S12. Crosstab table of rainfall class – prefecture.

Prefecture	Rainfall class		Total
	1 (< 70 mm)	2 (>70 mm)	
Arta	62	0	62
Preveza	3	50	53
Thesprotia	21	42	63
Total	86	92	**178**

Table S13. Crosstab table of rainfall class – variety.

Variety	Rainfall class		Total
	1 (< 70 mm)	2 (>70 mm)	
Amfissis	32	2	34
Koroneiki	14	12	26
Lianolia	7	56	63
Mesokarpos	7	7	14
Table olives	18	9	27
Kalamon	5	5	10
Agria	2	2	4
Total	86	92	**178**

Table S14. Results of Mann-Whitney U test between the two classes of temperature mean.

	Total phenolics	Oleocanthal (3)	Oleocein (4)	ligstroside aglycone (5)	oleuropein aglycone (6)	oleokoronal (7)	oleomissional (8)
Mann-Whitney U	4145	4235	3998	3487	3353	4049	4041
Asymp. Sig. (2-tailed)	0.590	0.424	0.912	0.169	0.077	0.796	0.802

Table S15. Interpretation of the correlation coefficient *r*

r	Interpretation
0.00–0.10	Negligible correlation
0.10–0.39	Weak correlation
0.40–0.69	Moderate correlation
0.70–0.89	Strong correlation
0.90–1.00	Very strong correlation

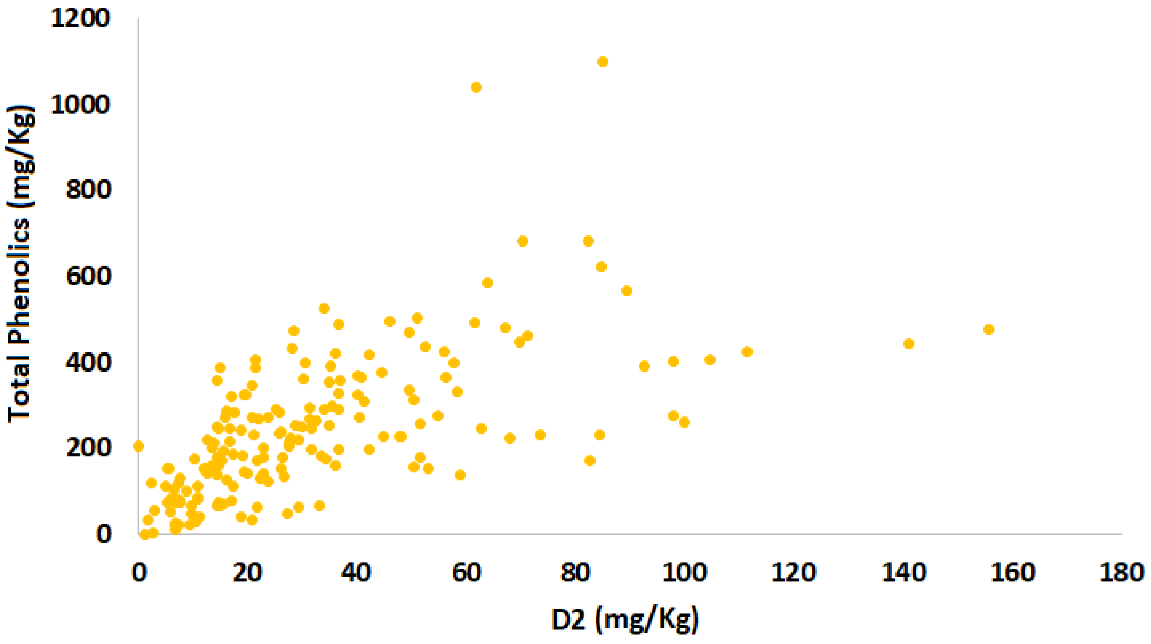

Figure S4. Total phenolics scatter plot versus index D2.

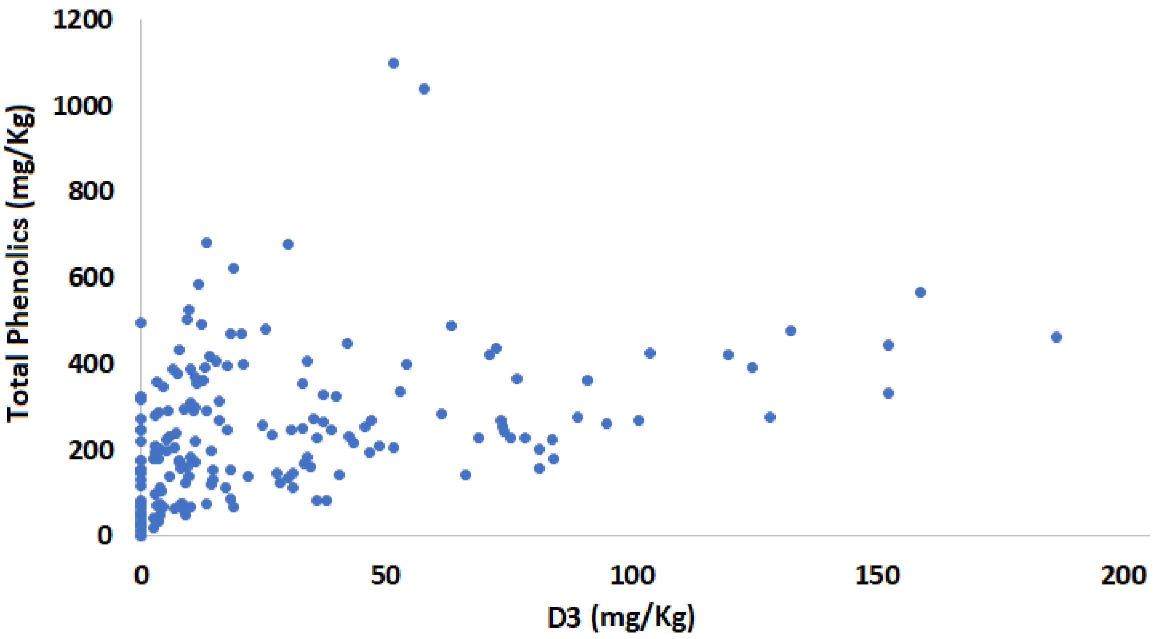

Figure S5. Total phenolics scatter plot versus index D3.

Table S16: The frequencies that were selected for suppression in the MSE method, at ppm.

Number of suppressed frequency	Frequency (in ppm)	Number of suppressed frequency	Frequency (in ppm)
**1**	0.87	**10**	1.65
**2**	0.88	**11**	2.00
**3**	0.89	**12**	2.02
**4**	1.26	**13**	2.03
**5**	1.27	**14**	2.33
**6**	1.31	**15**	2.36
**7**	1.32	**16**	5.33
**8**	1.62	**17**	5.34
**9**	1.63		

Table S17. Normality tests of mean amount of total phenolics among the olive variety.

	Kolmogorov-Smirnov			Shapiro-Wilk		
Variety	Statistic	N	Sig	Statistic	N	Sig
Amfissis	0.114	34	0.200	0.950	34	0.120
Koroneiki	0.132	26	0.200	0.963	26	0.458
Lianolia	0.056	63	0.200	0.976	63	0.262
Mesokarpos	0.127	14	0.200	0.964	14	0.791
Table olives	0.129	27	0.200	0.933	27	0.083
Kalamon	0.336	10	**0.002**	0.708	10	**0.001**
Agria	0.350	4	-	0.749	4	**0.038**

Table S18. Normality tests of mean amount of oleocanthal (**3**) among the olive variety.

	Kolmogorov-Smirnov			Shapiro-Wilk		
Variety	Statistic	N	Sig	Statistic	N	Sig
Amfissis	0.178	34	**0.008**	0.774	34	**< 0.001**
Koroneiki	0.175	26	**0.039**	0.935	26	0.103
Lianolia	0.059	63	0.200	0.977	63	0.277
Mesokarpos	0.199	14	0.137	0.900	14	0.113
Table olives	0.126	27	0.200	0.915	27	**0.029**
Kalamon	0.332	10	**0.003**	0.713	10	**0.001**
Agria	0.248	4	-	0.912	4	0.491

Table S19. Normality tests of mean amount of oleocein (**4**) among the olive variety.

	Kolmogorov-Smirnov			Shapiro-Wilk		
**Variety**	**Statistic**	**N**	**Sig**	**Statistic**	**N**	**Sig**
Amfissis	0.159	34	**0.029**	0.774	34	**< 0.001**
Koroneiki	0.138	26	0.200	0.935	26	0.210
Lianolia	0.089	63	0.200	0.977	63	**0.008**
Mesokarpos	0.211	14	0.090	0.900	14	**0.025**
Table olives	0.125	27	0.200	0.915	27	**0.044**
Kalamon	0.256	10	0.062	0.713	10	**0.009**
Agria	0.249	4	-	0.912	4	0.652

Table S20. Normality tests of mean amount of ligstroside aglycone (**5**) among the olive variety.

	Kolmogorov-Smirnov			Shapiro-Wilk		
**Variety**	**Statistic**	**N**	**Sig**	**Statistic**	**N**	**Sig**
Amfissis	0.196	34	**0.002**	0.857	34	**< 0.001**
Koroneiki	0.156	26	0.101	0.890	26	**0.010**
Lianolia	0.116	63	**0.035**	0.948	63	**0.010**
Mesokarpos	0.168	14	0.200	0.943	14	0.459
Table olives	0.266	27	**<0.001**	0.557	27	**< 0.001**
Kalamon	0.279	10	**0.026**	0.815	10	**0.022**
Agria	0.194	4	-	0.990	4	0.956

Table S21. Normality tests of mean amount of oleuropein aglycone (**6**) among the olive variety.

	Kolmogorov-Smirnov			Shapiro-Wilk		
**Variety**	**Statistic**	**N**	**Sig**	**Statistic**	**N**	**Sig**
Amfissis	0.202	34	**0.001**	0.835	34	**< 0.001**
Koroneiki	0.165	26	0.067	0.890	26	**0.009**
Lianolia	0.105	63	0.080	0.923	63	**< 0.001**
Mesokarpos	0.233	14	**0.037**	0.776	14	**0.003**
Table olives	0.224	27	**0.001**	0.709	27	**< 0.001**
Kalamon	0.246	10	0.089	0.879	10	0.125
Agria	0.259	4	-	0.910	4	0.484

Table S22. Normality tests of mean amount of oleokoronal (**7**) among the olive variety.

	Kolmogorov-Smirnov			Shapiro-Wilk		
**Variety**	**Statistic**	**N**	**Sig**	**Statistic**	**N**	**Sig**
Amfissis	0.184	34	**0.005**	0.835	34	**< 0.001**
Koroneiki	0.165	26	0.068	0.896	26	**0.013**
Lianolia	0.258	63	**<0.001**	0.710	63	**< 0.001**
Mesokarpos	0.328	14	**<0.001**	0.703	14	**< 0.001**
Table olives	0.191	27	**0.013**	0.748	27	**< 0.001**
Kalamon	0.343	10	**0.002**	0.645	10	**< 0.001**
Agria	0.178	4	-	0.985	4	0.933

Table S23. Normality tests of mean amount of oleomissional (**8**) among the olive variety.

	Kolmogorov-Smirnov			Shapiro-Wilk		
**Variety**	**Statistic**	**N**	**Sig**	**Statistic**	**N**	**Sig**
Amfissis	0.291	34	**<0.001**	0.567	34	**< 0.001**
Koroneiki	0.180	26	**0.030**	0.860	26	**0.002**
Lianolia	0.310	63	**<0.001**	0.547	63	**< 0.001**
Mesokarpos	0.344	14	**<0.001**	0.576	14	**< 0.001**
Table olives	0.193	27	**0.011**	0.846	27	**< 0.001**
Kalamon	0.472	10	**<0.001**	0.533	10	**< 0.001**
Agria	-	4	-	-	4	-

Table S24. Normality tests of mean amount of total phenolics among the harvest month.

	Kolmogorov-Smirnov			Shapiro-Wilk		
**Month**	**Statistic**	**N**	**Sig**	**Statistic**	**N**	**Sig**
October	0.214	20	**0.017**	0.734	20	**< 0.001**
November	0.072	99	0.200	0.926	99	**< 0.001**
December	0.115	41	**0.192**	0.846	41	**< 0.001**
January to March	0.198	18	**0.060**	0.872	18	**0.019**

Table S25. Normality tests of mean amount of oleocanthal (**3**) among the harvest month.

	Kolmogorov-Smirnov			Shapiro-Wilk		
**Month**	**Statistic**	**N**	**Sig**	**Statistic**	**N**	**Sig**
October	0.372	20	**< 0.001**	0.523	20	**< 0.001**
November	0.103	99	**0.011**	0.830	99	**< 0.001**
December	0.141	41	**0.038**	0.885	41	**< 0.001**
January to March	0.212	18	**0.031**	0.889	18	**0.037**

Table S26. Normality tests of mean amount of oleocein (**4**) among the harvest month.

	Kolmogorov-Smirnov			Shapiro-Wilk		
**Month**	**Statistic**	**N**	**Sig**	**Statistic**	**N**	**Sig**
October	0.137	20	0.200	0.925	20	0.123
November	0.111	99	**0.005**	0.956	99	**0.002**
December	0.225	41	**< 0.001**	0.609	41	**< 0.001**
January to March	0.234	18	**0.010**	0.752	18	**< 0.001**

Table S27. Normality tests of mean amount of ligstroside aglycone (**5**) among the harvest month.

	Kolmogorov-Smirnov			Shapiro-Wilk		
**Month**	**Statistic**	**N**	**Sig**	**Statistic**	**N**	**Sig**
October	0.188	20	0.062	0.816	20	**0.002**
November	0.090	99	**0.046**	0.893	99	**< 0.001**
December	0.172	41	**0.004**	0.863	41	**< 0.001**
January to March	0.178	18	0.138	0.911	18	0.089

Table S28. Normality tests of mean amount of total phenolics among the prefecture.

	Kolmogorov-Smirnov			Shapiro-Wilk		
**Prefecture**	**Statistic**	**N**	**Sig**	**Statistic**	**N**	**Sig**
Arta	0.067	62	0.200	0.959	62	**0.039**
Preveza	0.087	53	0.200	0.965	53	0.117
Thesprotia	0.104	63	0.086	0.845	63	**< 0.001**

Table S29. Normality tests of mean amount of oleocanthal (**3**) among the prefecture.

	Kolmogorov-Smirnov			Shapiro-Wilk		
**Prefecture**	**Statistic**	**N**	**Sig**	**Statistic**	**N**	**Sig**
Arta	0.134	62	**0.008**	0.959	62	**< 0.001**
Preveza	0.109	53	0.167	0.965	53	0.144
Thesprotia	0.164	63	**<0.001**	0.845	63	**< 0.001**

Table S30. Normality tests of mean amount of oleocein (**4**) among the prefecture.

	Kolmogorov-Smirnov			Shapiro-Wilk		
**Prefecture**	**Statistic**	**N**	**Sig**	**Statistic**	**N**	**Sig**
Arta	0.138	62	**0.005**	0.895	62	**< 0.001**
Preveza	0.089	53	0.200	0.935	53	**0.006**
Thesprotia	0.176	63	**<0.001**	0.907	63	**< 0.001**

Table S31. Normality tests of mean amount of ligstroside aglycone (**5**) among the prefecture.

	Kolmogorov-Smirnov			Shapiro-Wilk		
**Prefecture**	**Statistic**	**N**	**Sig**	**Statistic**	**N**	**Sig**
Arta	0.186	62	**<0.001**	0.861	62	**< 0.001**
Preveza	0.139	53	**0.012**	0.926	53	**0.003**
Thesprotia	0.126	63	**0.014**	0.903	63	**< 0.001**

Table S32. Normality tests of mean amount of oleuropein aglycone (**6**) among the prefecture.

	Kolmogorov-Smirnov			Shapiro-Wilk		
**Prefecture**	**Statistic**	**N**	**Sig**	**Statistic**	**N**	**Sig**
Arta	0.146	62	**0.002**	0.901	62	**< 0.001**
Preveza	0.143	53	**0.009**	0.876	53	**< 0.001**
Thesprotia	0.163	63	**<0.001**	0.808	63	**< 0.001**

Table S33. Normality tests of mean amount of oleokoronal (**7**) among the prefecture.

	Kolmogorov-Smirnov			Shapiro-Wilk		
**Prefecture**	**Statistic**	**N**	**Sig**	**Statistic**	**N**	**Sig**
Arta	0.156	62	**<0.001**	0.859	62	**< 0.001**
Preveza	0.285	53	**<0.001**	0.723	53	**< 0.001**
Thesprotia	0.273	63	**<0.001**	0.690	63	**< 0.001**

Table S34. Normality tests of mean amount of total phenolics among the altitude class.

	Kolmogorov-Smirnov			Shapiro-Wilk		
**Altitude class**	**Statistic**	**N**	**Sig**	**Statistic**	**N**	**Sig**
1 (≤ 150 m)	0.086	81	0.200	0.965	81	**0.026**
2 (> 150 m)	0.111	97	**0.005**	0.861	97	**< 0.001**

Table S35. Normality tests of mean amount of oleocanthal (**3**) among the altitude class.

	Kolmogorov-Smirnov			Shapiro-Wilk		
**Altitude class**	**Statistic**	**N**	**Sig**	**Statistic**	**N**	**Sig**
1 (≤ 150 m)	0.127	81	**0.002**	0.921	81	**< 0.001**
2 (> 150 m)	0.157	97	**<0.001**	0.708	97	**< 0.001**

Table S36. Normality tests of mean amount of oleocein (**4**) among the altitude class.

	Kolmogorov-Smirnov			Shapiro-Wilk		
Altitude class	Statistic	N	Sig	Statistic	N	Sig
1 (≤ 150 m)	0.147	81	**<0.001**	0.914	81	**< 0.001**
2 (> 150 m)	0.126	97	**<0.001**	0.901	97	**< 0.001**

Table S37. Normality tests of mean amount of ligstroside aglycone (**5**) among the altitude class.

	Kolmogorov-Smirnov			Shapiro-Wilk		
Altitude class	Statistic	N	Sig	Statistic	N	Sig
1 (≤ 150 m)	0.114	81	**0.011**	0.881	81	**< 0.001**
2 (> 150 m)	0.130	97	**<0.001**	0.873	97	**< 0.001**

Table S38. Normality tests of mean amount of oleuropein aglycone (**6**) among the altitude class.

	Kolmogorov-Smirnov			Shapiro-Wilk		
Altitude class	Statistic	N	Sig	Statistic	N	Sig
1 (≤ 150 m)	0.150	81	**<0.001**	0.856	81	**< 0.001**
2 (> 150 m)	0.183	97	**<0.001**	0.731	97	**< 0.001**

Table S39. Normality tests of mean amount of oleokoronal (**7**) among the altitude class.

	Kolmogorov-Smirnov			Shapiro-Wilk		
Altitude class	Statistic	N	Sig	Statistic	N	Sig
1 (≤ 150 m)	0.199	81	**< 0.001**	0.780	81	**< 0.001**
2 (> 150 m)	0.232	97	**< 0.001**	0.768	97	**< 0.001**

Table S40. Normality tests of mean amount of oleomissional (**8**) among the altitude class.

	Kolmogorov-Smirnov			Shapiro-Wilk		
Altitude class	Statistic	N	Sig	Statistic	N	Sig
1 (≤ 150 m)	0.139	81	**< 0.001**	0.821	81	**< 0.001**
2 (> 150 m)	0.063	97	0.200	0.931	97	**< 0.001**

Table S41. Normality tests of mean amount of total phenolics among the rainfall class.

	Kolmogorov-Smirnov			Shapiro-Wilk		
Altitude class	Statistic	N	Sig	Statistic	N	Sig
1 (< 70 mm)	0.086	86	0.200	0.965	86	**< 0.001**
2 (> 70 mm)	0.111	92	**0.005**	0.861	92	**< 0.001**

Table S42. Normality tests of mean amount of oleocanthal (**3**) among the rainfall class.

	Kolmogorov-Smirnov			Shapiro-Wilk		
Altitude class	Statistic	N	Sig	Statistic	N	Sig
1 (< 70 mm)	0.197	86	**< 0.001**	0.625	86	**< 0.001**
2 (> 70 mm)	0.095	92	**0.040**	0.791	92	**< 0.001**

Table S43. Normality tests of mean amount of oleocein (**4**) among the rainfall class.

	Kolmogorov-Smirnov			Shapiro-Wilk		
Altitude class	Statistic	N	Sig	Statistic	N	Sig
1 (< 70 mm)	0.147	86	**< 0.001**	0.864	86	**< 0.001**
2 (> 70 mm)	0.085	92	0.099	0.941	92	**< 0.001**

Table S44. Normality tests of mean amount of ligstroside aglycone (**5**) among the rainfall class.

	Kolmogorov-Smirnov			Shapiro-Wilk		
Altitude class	Statistic	N	Sig	Statistic	N	Sig
1 (< 70 mm)	0.143	86	**<0.001**	0.891	86	**< 0.001**
2 (> 70 mm)	0.132	92	**<0.001**	0.862	92	**< 0.001**

Table S45. Normality tests of mean amount of oleuropein aglycone (**6**) among the rainfall class.

	Kolmogorov-Smirnov			Shapiro-Wilk		
Altitude class	Statistic	N	Sig	Statistic	N	Sig
1 (< 70 mm)	0.177	86	**<0.001**	0.747	86	**< 0.001**
2 (> 70 mm)	0.163	92	**<0.001**	0.807	92	**< 0.001**

Table S46. Normality tests of mean amount of oleokoronal (**7**) among the rainfall class.

	Kolmogorov-Smirnov			Shapiro-Wilk		
Altitude class	Statistic	N	Sig	Statistic	N	Sig
1 (< 70 mm)	0.173	86	**< 0.001**	0.840	86	**< 0.001**
2 (> 70 mm)	0.268	92	**< 0.001**	0.681	92	**< 0.001**

Table S47. Normality tests of mean amount of oleomissional (**8**) among the rainfall class.

	Kolmogorov-Smirnov			Shapiro-Wilk		
Altitude class	Statistic	N	Sig	Statistic	N	Sig
1 (< 70 mm)	0.259	86	**< 0.001**	0.671	86	**< 0.001**
2 (> 70 mm)	0.297	92	**< 0.001**	0.596	92	**< 0.001**

Table S48. Normality tests of mean amount of total phenolics among the temperature class.

	Kolmogorov-Smirnov			Shapiro-Wilk		
**Temperature class**	**Statistic**	**N**	**Sig**	**Statistic**	**N**	**Sig**
1 (< 16.5 °C)	0.116	88	**0.005**	0.880	88	**< 0.001**
2 (≥ 16.5 °C)	0.083	90	0.168	0.898	90	**< 0.001**

Table S49. Normality tests of mean amount of oleocanthal (**3**) among the temperature class.

	Kolmogorov-Smirnov			Shapiro-Wilk		
**Temperature class**	**Statistic**	**N**	**Sig**	**Statistic**	**N**	**Sig**
1 (< 16.5 °C)	0.144	88	**< 0.001**	0.762	88	**< 0.001**
2 (≥ 16.5 °C)	0.128	90	**< 0.001**	0.749	90	**< 0.001**

Table S50. Normality tests of mean amount of oleocein (**4**) among the temperature class.

	Kolmogorov-Smirnov			Shapiro-Wilk		
**Temperature class**	**Statistic**	**N**	**Sig**	**Statistic**	**N**	**Sig**
1 (< 16.5 °C)	0.138	88	**< 0.001**	0.875	88	**< 0.001**
2 (≥ 16.5 °C)	0.134	90	**< 0.001**	0.925	90	**< 0.001**

Table S51. Normality tests of mean amount of ligstroside aglycone (**5**) among the temperature class.

	Kolmogorov-Smirnov			Shapiro-Wilk		
**Temperature class**	**Statistic**	**N**	**Sig**	**Statistic**	**N**	**Sig**
1 (< 16.5 °C)	0.119	88	**0.003**	0.917	88	**< 0.001**
2 (≥ 16.5 °C)	0.136	90	**<0.001**	0.820	90	**< 0.001**

Table S52. Normality tests of mean amount of oleuropein aglycone (**6**) among the temperature class.

	Kolmogorov-Smirnov			Shapiro-Wilk		
**Temperature class**	**Statistic**	**N**	**Sig**	**Statistic**	**N**	**Sig**
1 (< 16.5 °C)	0.170	88	**<0.001**	0.762	88	**< 0.001**
2 (≥ 16.5 °C)	0.164	90	**<0.001**	0.843	90	**< 0.001**

Table S53. Normality tests of mean amount of oleokoronal (**7**) among the temperature class.

	Kolmogorov-Smirnov			Shapiro-Wilk		
**Temperature class**	**Statistic**	**N**	**Sig**	**Statistic**	**N**	**Sig**
1 (< 16.5 °C)	0.222	88	**< 0.001**	0.789	88	**< 0.001**
2 (≥ 16.5 °C)	0.213	90	**< 0.001**	0.760	90	**< 0.001**

Table S54. Normality tests of mean amount of oleomissional (**8**) among the temperature class.

	Kolmogorov-Smirnov			Shapiro-Wilk		
Temperature class	Statistic	N	Sig	Statistic	N	Sig
1 (< 16.5 °C)	0.287	88	**< 0.001**	0.606	88	**< 0.001**
2 (≥ 16.5 °C)	0.269	90	**< 0.001**	0.661	90	**< 0.001**

References

1. Tsiafoulis, C.G.; Liaggou, C.; Garoufis, A.; Magiatis, P.; Roussis, I.G. Nuclear Magnetic Resonance Analysis of Extra Virgin Olive Oil: Classification through Secoiridoids. *J. Sci. Food Agric*. **2023**, *104*, 1992-2005, doi:10.1002/jsfa.13139.
2. Gottstein, V.; Müller, M.; Günther, J.; Kuballa, T.; Vetter, W. Direct 1H NMR Quantitation of Valuable Furan Fatty Acids in Fish Oils and Fish Oil Fractions. *J. Agric. Food Chem.* **2019**, *67*, 11788–11795, doi:10.1021/acs.jafc.9b04711.
